# Bio-based polycarbonate as synthetic toolbox

**DOI:** 10.1038/ncomms11862

**Published:** 2016-06-15

**Authors:** O. Hauenstein, S. Agarwal, A. Greiner

**Affiliations:** 1 Macromolecular Chemistry II and Center for Colloids and Interfaces, University of Bayreuth, Universitätsstrasse 30, 95440 Bayreuth, Germany

## Abstract

Completely bio-based poly(limonene carbonate) is a thermoplastic polymer, which can be synthesized by copolymerization of limonene oxide (derived from limonene, which is found in orange peel) and CO_2_. Poly(limonene carbonate) has one double bond per repeating unit that can be exploited for further chemical modifications. These chemical modifications allow the tuning of the properties of the aliphatic polycarbonate in nearly any direction. Here we show synthetic routes to demonstrate that poly(limonene carbonate) is the perfect green platform polymer, from which many functional materials can be derived. The relevant examples presented in this study are the transformation from an engineering thermoplastic into a rubber, addition of permanent antibacterial activity, hydrophilization and even pH-dependent water solubility of the polycarbonate. Finally, we show a synthetic route to yield the completely saturated counterpart that exhibits improved heat processability due to lower reactivity.

## Abstract (web summary)

With growing concerns over the sustainability of petroleum-based polymers, poly(limonene carbonate) is offered as a renewable alternative. Here, Greiner and co-workers have managed to tune the properties of poly(limonene carbonate) by carrying out several different chemical modifications.

## Graphical abstract

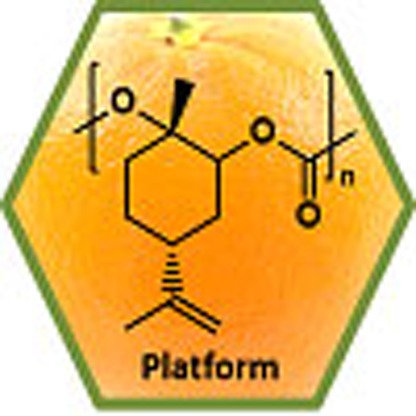

The petroleum-based plastics industry is facing two major challenges. On the one hand, there is the urgent environmental problem of pollution of the ocean with about five million tonnes of plastic waste per year^1^,^2^. On the other hand, there is a natural limitation of petroleum resources, which eventually leads to a running out of oil and natural gas within this century^3^. To overcome these limitations, efforts are directed towards the development of degradable polymers^4^,^5^ and the use of bio-based monomers^6^,^7^,^8^, respectively. Sometimes, both classes are combined, that is, the polymer is bio-based and biodegradable, for example, poly(lactic acid) or polyhydroxyalkanoates^9^,^10^,^11^. In other instances, the polymer can be assigned either to the class of bio-based non-degradable plastics, such as bio-polyethylene or bio-poly(ethylene terephthalate), or to the class of biodegradable petro-based plastics, for example, poly(*ɛ*-caprolactone) or poly(butylene adipate-co-terephthalate)^8^,^12^. However, even for a material that is assigned to both classes—as for poly(lactic acid)—the origin of the bio-based monomers is questionable, as lactic acid is derived from glucose, which is again derived from corn starch. The latter is also an important food resource and, as such, in competition with the use as precursor for the conversion into plastics. In contrast, limonene—a doubly unsaturated terpene—is a bio-based non-food resource, which is mainly derived from the peel of citrus fruits^13^,^14^,^15^. As the major component of orange oil (>90%), it is an abundantly available side product of the orange industry, produced in amounts of roughly 500 kt per year^16^. Its versatility as a monomer is reflected by the great variety of polymers that are derived from limonene^13^,^17^,^18^,^19^. In 2004, Coates and colleagues^20^ reported the elegant metal-catalysed conversion of its oxidation product limonene oxide (LO) with CO_2_, to give a low-molecular-weight poly(limonene carbonate) (PLimC) (Fig. 1). An Al(III)-based catalyst was recently found to incorporate not only the *trans*- but also the *cis*-isomer of LO, which is an important step towards higher conversions^21^.

Inspired by the work of Coates *et al.*^20^, we modified the copolymerization to yield high-molecular-weight (>100 kDa) PLimC in kilogram quantities^22^ with a glass transition temperature of 130 °C, higher transparency than bisphenol-A polycarbonate (bisphenol A polycarbonate (BPA PC); 94 versus 89%) and improved mechanical properties compared with the petro-based counterpart poly(cyclohexene carbonate) (strain at break of 2 versus 15% for PLimC)^23^. The amorphous thermoplastic still possesses one double bond per repeating unit. This suggests a broad range of modifications to tune the properties in almost any direction. Thus, we consider PLimC a platform, from which countless functional materials can be derived. To support this statement, we give here relevant examples of straightforward addition reactions, that is, thiol-ene click chemistry^24^, acid-catalysed electrophilic addition and metal-catalysed hydrogenation. The first two are conducted as polymer-analogous reactions, whereas the latter involves modification of the pre-monomer limonene. The manipulations lead to dramatic changes in the property profile of the engineering thermoplastic PLimC, including a transformation into a rubbery material, antibacterial activity, increased hydrophilicity or even water solubility and, last but not least, improved melt processability.

## Results

### Modification of unsaturated PLimC

**Figure 2:**
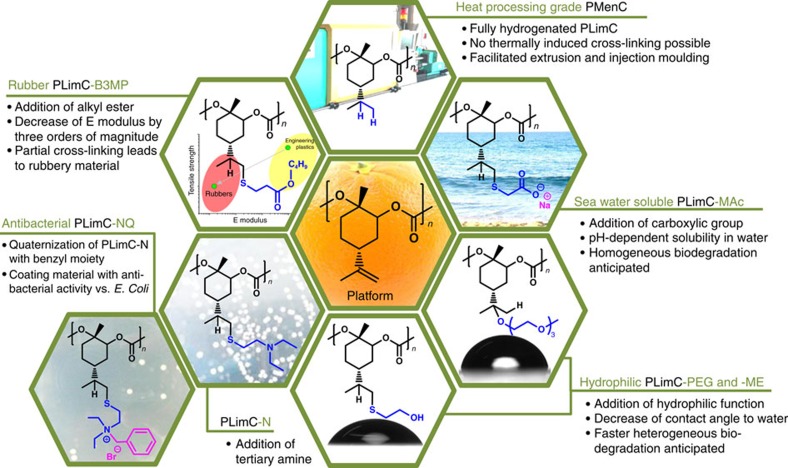
The valorization of PLimC. The versatility of the platform polymer PLimC is illustrated in this hexagon cluster. The double bond is used for addition reactions, to induce dramatic changes of the properties of PLimC. The addition of an alkyl ester (PLimC-B3MP) leads to a drop of *T*_g_ by 120 °C and of its Young's modulus by three orders of magnitude to give a PLimC rubber. The addition of a carboxylic group (PLimC-MAc) yields a pH-dependent solubility in water, whereas the functionalization with hydroxyl (PLimC-ME) or polyethylene glycol (PLimC-PEG) groups results in a higher hydrophilicity. The attachment of a tertiary amine (PLimC-N) and subsequent quaternization with an aromatic moiety (PLimC-NQ) leads to antibacterial activity against *E. coli*. Another possibility is the complete hydrogenation of the double bond to give PMenC, a superior material for heat processing.

The valorization of the platform polymer PLimC is illustrated in Fig. 2. When butyl-3-mercaptopropionate (B3MP) is used, an enormous change in mechanical properties is achieved. This leads to a transformation of the high-*T*_g_ thermoplastic into rubbery PLimC-B3MP with a nearly three orders of magnitude decreased Young's modulus. Furthermore, this chemistry is also applied to transform PLimC into an antibacterial material, by covalently attaching a tertiary amine to the backbone (PLimC-N) and subsequent quaternization with an aromatic moiety (PLimC-NQ). The antibacterial activity of PLimC-NQ was successfully tested against *Escherichia coli* bacteria. Another aspect is the hypothetical biodegradability of PLimC, which would be expected for an aliphatic PC, as it was shown for others^25^,^26^,^27^. Initial composting studies on PLimC revealed no degradation after prolonged exposure at elevated temperatures. This rather shows the high biostability of the material, which is also desirable for many applications. Here we present two major synthetic routes, to tune the degradation behaviour of the rigid and hydrophobic polymer, that is, either an acid-catalysed electrophilic addition of poly(ethylene glycol)monomethyl ether (PEG-3-OH) resulting in PLimC-PEG or thiol-ene chemistry with mercaptoethanol (ME) to give PLimC-ME or mercaptoacetic acid (MAc) to yield PLimC-MAc, respectively. For the latter, this eventually even leads to pH-dependent water solubility, that is, the material dissolves readily in basic environment. Apart from the above-mentioned addition reactions, the synthetic route to the fully hydrogenated PC poly(menthene carbonate) (PMenC) is also reported for the first time. The saturated PC might be a viable choice for replacing PLimC in thermal processing, as no cross-linking can occur. Thus, starting from the bio-based platform polymer PLimC, we could introduce antibacterial activity (ideal as coating material)^28^,^29^, elastomeric behaviour, hydrophilization, water solubility (both should accelerate biodegradation) and, in fact, inertness by hydrogenation to improve processing.

### The transformation into elastic PLimC

**Figure 3:**
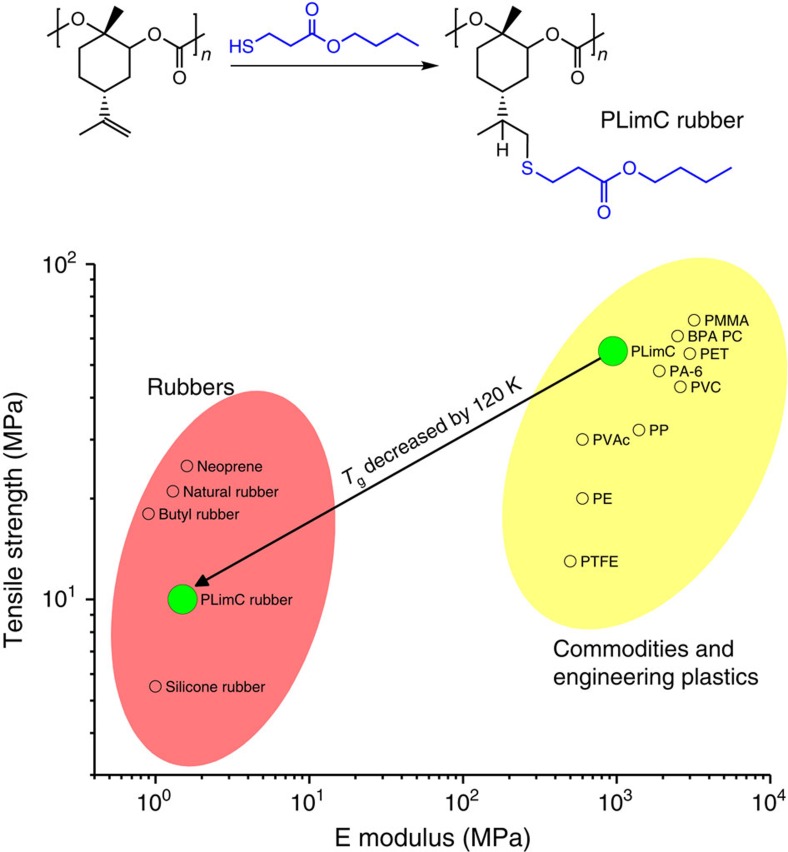
The transformation into elastic PLimC. The schematic illustrates the functionalization of PLimC with B3MP to give PLimC-B3MP, that is, a PLimC rubber. The Young's moduli of various engineering plastics, commodities (both yellow region) and rubbers (red region) are plotted against their tensile strength. The materials PLimC and PLimC rubber are highlighted as green circles, showing the dramatic change of mechanical properties on functionalization of pure PLimC with B3MP.

The enormous versatility of PLimC is reflected in an experiment, where the thiol-functionalized ester B3MP—the pure ester is found in many fruits^30^—is clicked to the double bond in nearly quantitative yield (schematic in Fig. 3 and Supplementary Figs 1–3). The *T*_g_ of PLimC lies at 130 °C, rendering it a typical engineering thermoplastic such as polyamide, poly(ethylene terephthalate), BPA PC and so on (yellow region in Ashby plot of Fig. 3 and Supplementary Table 1)^31^. The covalently attached butyl ester B3MP changes the thermal and hence the mechanical properties dramatically, that is, the *T*_g_ drops to 5 °C (Table 1, Supplementary Table 2 and Supplementary Fig. 4). Tensile testing of the new material revealed its high maximum elongation *ɛ* combined with a low Young's modulus and tensile strength *σ*_s_, respectively. PLimC-B3MP with 0–2% residual double bonds in the backbone was prepared by variation of the reaction time (Supplementary Fig. 5). Curing the unsaturated polymers at 100 °C for 5 h renders the cross-linked samples insoluble, whereas the mechanical properties can be adjusted (Supplementary Fig. 6; for a detailed discussion on the curing process, see Supplementary Discussion). These observations combined with the elasticity (Supplementary Fig. 7) suggest a transition from the engineering thermoplastics region to the rubber region, that is, the thermoplastic PLimC has become a PLimC rubber (PLimC-B3MP, red region in Fig. 3)^32^.

This transition enables the application of the bio-based material in completely new areas, where elasticity and softness are required. Furthermore, we introduced a short alkyl chain ester into the repeating unit of PLimC. Addition of longer alkyl chains potentially leads to *T*_g_ values well below 0 °C, which is another very important parameter to tune the performance of the resulting rubber. This is an example of the transformation of PLimC into a completely new material by simple polymer analogous click chemistry, while keeping the material based on natural resources. Further studies will focus on the reduction of *T*_g_ and the control of mechanical properties to expand the coverage of the Ashby plot.

### The transformation into antibacterial PLimC

**Figure 4:**
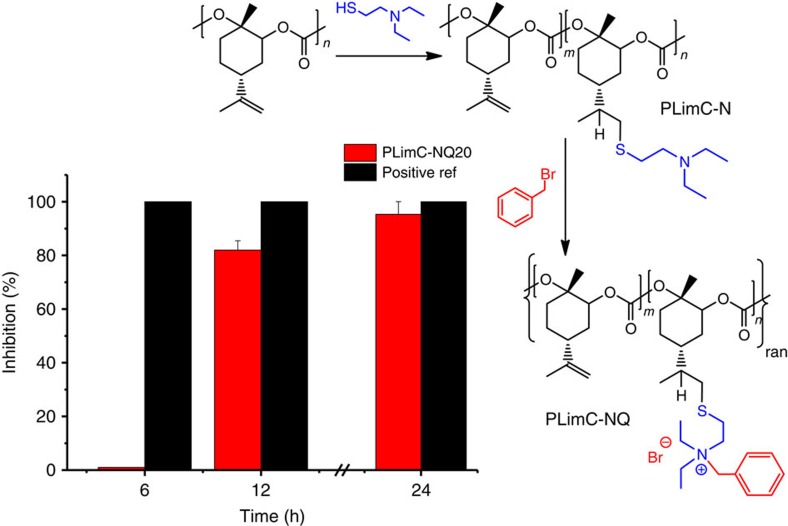
The transformation into antibacterial PLimC. Schematic of the functionalization of PLimC with a tertiary amine (PLimC-N) and the subsequent quaternization with a benzyl moiety (PLimC-NQ). In the column diagram, the bacterial inhibition performances of PLimC-NQ20 and a positive reference material (polyhexamethylene guanidine hydrochloride, PHMG) relative to pure PLimC in a shaking flask experiment; tested on *E. coli* bacteria in buffer solution with polymer films (20 mg ml^−1^) at 20 °C are illustrated. The inhibition was calculated by determination of the cfu of tenfold diluted dispersions spread on agar plates (Supplementary Table 7).

In a recent publication we stated the thermal properties of PLimC, that is, a *T*_g_ of 130 °C and a 5% decomposition temperature (*T*_5%_) of 240 °C, resulting in a rather narrow window for processing^22^. As an alternative to thermal processing, that is, extrusion or injection moulding, the employment of a PLimC solution for the application as a coating is self-evident. The high transparency and good scratch resistance are very promising properties that should yield high-value materials in combination with an extra functionality. We picked one out of many possible functionalizations, to show how PLimC can be transformed into an antibacterial material by rather simple means. The strategy involves the addition of a tertiary amine to PLimC via thiol-ene click chemistry (PLimC-N) and the subsequent quaternization of the amine with an aryl halide (PLimC-NQ) (Supplementary Figs 8–14 and Supplementary Tables 3 and 4). The functionalization was performed with different degrees of functionalization (DFs), although keeping it below 70% to keep the material insoluble in water. Resistance to water is of major importance, to make the material applicable as coatings in everyday life, where contact with water is inevitable. On the other hand, antibacterial activity rises exponentially, if the polymer—or part of it—is water soluble, as interaction of the charged amine with the bacteria's membrane is facilitated^33^. Therefore, a sample with 20% quaternized amine (PLimC-NQ20) was investigated, which does not disintegrate in contact with water and still shows antibacterial activity by inhibiting bacterial growth. For the evaluation of the antibacterial properties of the coating, films were placed in *E. coli* suspensions and the concentration of the Gram-negative bacteria was assessed after 0, 6, 12, 24 and 48 h (Supplementary Tables 5–7). Compared with pure PLimC, PlimC-NQ20 exhibited a strong inhibitory effect on bacteria growth after 24 h. The ratio of killed bacteria relative to PLimC samples is illustrated in Fig. 4. The inhibitory effect for the positive reference material polyhexamethylene guanidine hydrochloride is detected already after 6 h, when all bacteria were killed. The charged PLimC samples are less active. This is not surprising, as they are in condensed state and not dissolved such as polyhexamethylene guanidine hydrochloride. Still, the antibacterial activity could be observed after 12 h of contact with the bacteria suspension, indicating a successful valorization of PLimC into antibacterial PLimC.

Here, as proof of principle, we can demonstrate that PLimC is readily transformable into a material with antibacterial activity by rather simple and cost-effective means. We would assume though that lots of parameters are still to be optimized, that is, DF, length of spacer between thiol and amine, nature of the alkyl or aryl moiety on the amine and random distribution of quaternized amine along the backbone of the polymer versus block copolymer structure. Furthermore, the types of bacteria have to be selected in respect of the targeted application.

### The transformation into hydrophilic/water-soluble PLimC

**Figure 5:**
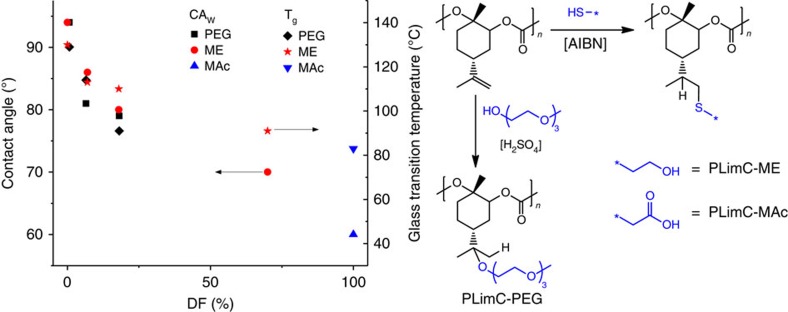
The transformation into hydrophilic/water-soluble PLimC. Dependency of contact angle to water and *T*_g_ of a PLimC film on the degree of functionalization with PEG-3-OH (black), ME (red) or MAc (blue), respectively. The schematic of the functionalization is shown for the thiol-ene addition of ME and MAc to give PLimC-ME and PLimC-MAc, respectively, and the electrophilic addition of PEG-3-OH to give PLimC-PEG.

The idea to render PLimC more hydrophilic was born, when studies on the degradation behaviour of pure PLimC in highly active compost at 60 °C (positive reference poly(L-lactic acid) disintegrated within 1 week) had been stopped after 60 days, because no change, neither in the outer appearance nor in molecular weight, had been observed. The rather substantial biostability of this aliphatic PC is most probably explained by the three facts about PLimC. First of all, it has a very rigid backbone, resulting in a high *T*_g_ of 130 °C, which is ∼100 °C higher than that of readily biodegradable poly(propylene carbonate)^34^,^35^. The rigidity of the backbone prevents the polymer chain segments from moving and, therefore, no exposure of the carbonate groups to enzymes/bacteria is possible. Second, the polymer carries a very bulky ‘side group', consisting of the cyclohexane ring connected to an isopropylene group and another methyl moiety vicinal to the carbonate. Thus, even if there are some exposed chains on the surface of such a film, the carbonate group is shielded against any attacking species. Eventually, PLimC is very hydrophobic, which is represented by its contact angle to water (CA_W_) of 94°. This prevents not only enzymes from penetrating the polymer but also water. Hence, acid or basic hydrolysis—usually the major breakdown mechanism for long polymer chains—is inhibited; thus, nearly no fragmentation of PLimC for further degradation takes place. In consideration of the underlying circumstances, the hydrophilization of PLimC was assumed to be a likely enabler for biodegradation. Three different strategies were employed to achieve hydrophilic PLimC: two of them involve the well-known thiol-ene chemistry with ME (Supplementary Fig. 15 and Supplementary Table 8) or MAc as thiols, respectively (Supplementary Fig. 16)^36^,^37^,^38^,^39^,^40^. The other strategy is—an even simpler—acid-catalysed electrophilic addition of PEG-3-OH to the double bond (Supplementary Fig. 17 and Supplementary Table 9). The latter can also be acknowledged as a green reaction, hence keeping the bio-based character of PLimC, while grafting polar functionality. Indeed, the higher the DF, the smaller CA_W_ becomes. Up to 18% conversion of the double bond was achieved by this electrophilic addition; however, as it is acid catalysed, hydrolysis of the carbonate is an immanent side reaction, which eventually breaks down the polymer chains (Supplementary Fig. 18). Thus, reaction times were kept short and a great excess of PEG-3-OH was maintained. The contact angle could be decreased below 80° with this technique. An even stronger decrease of the CA_W_ could be achieved by radically adding ME to PLimC. Compared with the acid-catalysed addition, the advantage of thiol-ene chemistry is the absence of hydrolytic side reactions. Therefore, higher DFs are easily accessible. Here a DF of 70% resulted in a CA_W_ of 70°. To hydrophilize the polymer even further, PLimC was functionalized with MAc, whereas 100% attachment of the acid yields a polymer with a CA_W_ of 60°. A side effect of all above-mentioned modifications is the decrease of *T*_g_ with increasing DF (Fig. 5 and Supplementary Figs 19–21). In terms of degradability, this should further promote the breakdown of the polymer. The PEG- and ME-modified PLimCs were assessed regarding their degradation behaviour in acidic (pH 4), basic (pH 9) and enzymatic environment (esterase) at elevated temperatures (37 °C). Within the timespan of 4 weeks, no degradation characteristics, that is, loss of mass, drop in molecular weight or surface alterations, could be observed (Supplementary Fig. 22 and Supplementary Table 10). These observations suggest that neither a bulk nor a surface erosion process is taking place for the samples in those environments on the time scale measured. Keeping in mind that hydrophilicity and the chain flexibility were augmented, but steric shielding of the carbonate group was unchanged (or even higher due to addition reactions), we noticed that the overall stability versus hydrolysis and/or enzymatic attack is still too high. However, on longer time scales, the backbone of PLimC is anticipated to be much more labile than that of a polyolefin and degradation should take place eventually. This makes it an interesting choice, wherever good stability against hydrolysis during application is required, but eventual disintegration on a reasonable timescale is desired.

The functionalization of PLimC with acid functionality renders the material not only hydrophilic (CA_W_=60°) but also pH responsive. A film of PLimC-MAc (DF=100%) placed into a pH >8 buffer solution will dissolve within minutes. In the dissolved state, hydrolysis is of course highly accelerated compared with the condensed state. Therefore, the material would quickly disintegrate in seawater, which is usually slightly basic, and chain scission could readily occur. The exact degradation mechanism is yet to be studied, but this polymer could contribute in reducing the waste accumulation in the oceans^1^.

### The saturation of PLimC

**Figure 6:**
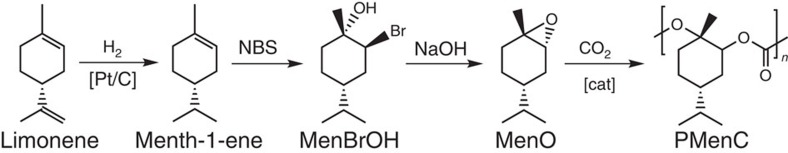
The saturation of PLimC. The synthetic route to PMenC starts from the metal-catalysed hydrogenation of limonene, followed by the stereoselective epoxidation of Men via its bromohydrin (MenBrOH) to *trans*-MenO and the subsequent copolymerization with CO_2_ to give the saturated PC PMenC.

Apart from the functionalization of PLimC, of course, it is also possible to hydrogenate the double bond, to render it unreactive, when it is heat processed or stored for a prolonged period of time. In contrast to the aforementioned modifications, this is not a polymer-analogous reaction, but the manipulation is performed on the pre-monomer (R)-limonene. Indeed, this hydrogenation is very regioselective, when a heterogeneous catalyst such as Pt on charcoal is used. Hence, a quantitative conversion to menth-1-ene (Men) can be achieved in reasonable time, whereas separation from the catalyst/carrier material is easy. We used the *N*-bromosuccinimide route for epoxidation (see Fig. 6) of Men to menthene oxide (MenO), as it is an established reaction for the stereo- and regio-selective epoxidation of limonene^22^. The route involves the formation of the bromohydrin MenBrOH and the subsequent ring closure in a basic medium to yield MenO. The conversion of the monomer MenO and pre-monomers Men and MenBrOH, respectively, were monitored by gas chromatography (GC) analysis (Supplementary Figs 23–25).

As the catalyst for the production of PMenC by copolymerization with CO_2_ is also selective towards the *trans*-isomer of MenO (Supplementary Figs 26–28), we still recommend taking the detour via the bromohydrin, but plan to look into new reactions to perform a more economical oxidation of menthene. The properties of PMenC are very similar to PLimC, as both are high-*T*_g_ amorphous PCs (Supplementary Fig. 29). The only, and of course anticipated, difference is the inability of the polymer to cross-link or to perform any undesired oxidation reactions at elevated temperatures. For PLimC, the addition of antioxidants, that is, butylated hydroxytoluene derivatives, helps to reduce those side reactions, but for PMenC no additives are needed. Indeed, a better processablilty, that is, extrusion and injection moulding, and a prolonged ultraviolet stability are anticipated. Both give extra value to this polymer. A combination of both MenO and LO for copolymerization with CO_2_ is also possible, resulting in a defined number of available double bonds for postmodification reactions as shown above.

## Discussion

In summary, we could show the huge versatility of the green platform polymer PLimC. The valorization was achieved either by polymer analogous thiol-ene chemistry and acid-catalysed electrophilic addition, or by metal-catalysed hydrogenation of the pre-monomer. Thiol-ene chemistry proved to be the most versatile technique, adding not only hydrophilicity or pH-dependent solubility, but also antibacterial activity by functionalization with quaternary amines. Even a transformation of the high-*T*_g_ thermoplastic PLimC into a rubbery material could be achieved by addition of a thiol. Not as adaptable is the acid-catalysed electrophilic addition of alcohols, although it proves to be more economical, as no costly functional thiols (employed in great excess) but only PEG and sulphuric acid are needed. Keeping the lability of the backbone of the aliphatic PC in mind, we note that this method is limited to short reaction times and hence only partial functionalization can be achieved. A quantitative conversion is possible for the regioselective hydrogenation of limonene, resulting in fully saturated PMenC after a few steps. This PC is oxidation resistant and thus exhibits improved processability for extrusion and injection moulding. The valorization of PLimC significantly broadens the range of applications, as PLimC-NQ is a viable antibacterial coating material and PLimC-ME, PLimC-PEG and PLimC-MAc could be employed as packaging materials with tuneable degradation/dissolution mechanism.

## Methods

### Instrumentation and characterization

NMR spectra were recorded on a Bruker AMX-300 operating at 300 MHz. Chemical shifts *δ* are indicated in parts per million with respect to residual solvent signals. Thermogravimetric analysis was performed on a Netzsch TG 209 F1 Libra and differential scanning calorimetry on a Mettler Toledo DSC 821c, both at a heating rate of 10 K min^−1^ under N_2_ atmosphere. Infrared spectra of solids were recorded with an attenuated total reflection unit of a Digilab Excalibur FTS-3000. GC analysis was performed on a Shimadzu QP-5050 with N_2_ as the carrier gas (temperature profile for GC studies: start at 40 °C hold for 5 min, heating to 80 °C with 5 °C min^−1^, heating to 120 °C with 3 °C min^−1^ and hold for 3 min, heating to 300 °C with 30 °C min^−1^). Relative molecular weights and dispersities were determined by gel permeation chromatography on an Agilent 1200 system with chloroform as the eluent and polystyrene as the calibration standard. A Hazemeter BykGardner Haze-Gard Plus and a ultraviolet–visible spectrometer V-670 (JASCO) were employed for the testing of optical properties of solvent cast PLimC films having a thickness between 100 and 400 μm. A Zwick/Roell Z0.5 test equipment with testXpert II software was employed for the tensile testing. The tests were performed at 21 °C and a relative humidity of 20%. The strain rate was set to 5 mm min^−1^, to test the tensile properties of cast polymer films that were die-cut into specimen (dumb-bell shaped) having a width of 2 mm, a length of 20 mm and a thickness of 100–200 μm. A BYK Pencil Hardness Tester and Derwent Graphic pencils were used to determine pencil hardness.

### Synthetic procedures

All synthetic manipulations were carried out under exclusion of air in dry conditions, if not otherwise stated. The acid-catalysed electrophilic addition and the thiol-ene chemistry were carried out as polymer analogous reactions. The hydrogenation of the exo double bond of limonene was performed on the pre-monomer, which was subsequently epoxidized and copolymerized with CO_2_, to give the PC PMenC. A detailed description of the synthetic procedures is given in the Supplementary Methods.

### Degradation tests in composting environment

Composting tests were performed on cast films of PLimC (*M*_n_=54.0 kDa, *Ð*=1.11) of 200 μm thickness fixed in slide mounts. The 3-month matured compost was supplied by an industrial composting plant (Mistelbach) and directly used for PLimC burial tests. Poly(L-lactic acid) (NatureWorks) was used as the positive reference material. During the test, the temperature was kept at 60 °C and the container was vented every 2 days (every 3 days after the first 2 weeks) and humidified if necessary (humidity was estimated by weighing of the container).

### Degradation tests in enzymatic environment

The enzymatic tests were performed on cast films of PLimC, PLimC-ME (100% functionalized with ME) and BPA PC (reference material) having a thickness of 100 μm, which were cut into 40 mg pieces. As media water, pH 4 buffer, pH 9 buffer and an esterase in pH 9 buffer (Esterase EL-01, triacylglycerol lipase, ASA Spezialenzyme GmbH, one part of enzyme suspension mixed with four parts of buffer solution, replaced after 10 days) were selected to test the polymer samples' stability against. At a temperature of 37 °C, the samples were shaken in a mechanical shaker (50 r.p.m.) in 40 ml glass containers, which were filled to 75%. The mass loss (balance) and molecular weight (gel permeation chromatography) change were analysed after 3, 10 and 21 days in triplicate for each sample.

### Antibacterial activity tests

*E. coli* (DSM no. 1077, K12 strain 343/113, DSMZ), as a Gram-negative test organism, was used to evaluate the antibacterial activity. We have chosen the Gram-negative bacteria *E. coli* for the antibacterial activity tests of PLimC-NQ, to evaluate the general activity of the polymer towards a very common bacterium. CASO-Boullion was used as nutrient for the *E. coli* (30 g l^−1^ in distilled water for liquid nutrient; 15 g l^−1^ agar-agar in addition for nutrient agar plates). The strain was preserved on nutrient agar plates and liquid cultures were grown by inoculation of liquid nutrient with a single bacteria colony using an inoculation loop. The inoculated broth was incubated with shaking at 37 °C until the optical density at 578 nm had risen to 0.125, indicating a cell density of 10^7^–10^8^ cfu ml^−1^. To obtain the final bacterial suspensions, the inoculated broth was diluted with buffer solution (phosphate-buffered solution, concentration of phosphate ions=12 mM, pH 7.4) to an approximate cell density of 10^5^ cfu ml^−1^. The antibacterial activity was determined by the shaking flask method: polymer films with a mass of 40 mg and a thickness of 100 μm were incubated with 2 mL of bacteria suspension at ambient temperature in microcentrifuge tubes with contact times of 6, 12 and 24 h. After the defined time intervals, 100 μl specimens were drawn and spread on nutrient agar plates. After incubation at 37 °C for 24 h, colonies were counted and the reduction was calculated relative to the unfunctionalized PLimC sample.

### Data availability

The authors declare that the data supporting the findings of this study are available within the article (and its Supplementary Information files).

## Additional information

**How to cite this article:** Hauenstein, O. *et al.* Bio-based polycarbonate as synthetic toolbox. *Nat. Commun.* 7:11862 doi: 10.1038/ncomms11862 (2016).

## Supplementary Material

Supplementary Information Supplementary Figures 1-29, Supplementary Tables 1-10, Supplementary Discussion, Supplementary Methods and Supplementary References

## Figures and Tables

**Figure 1:**
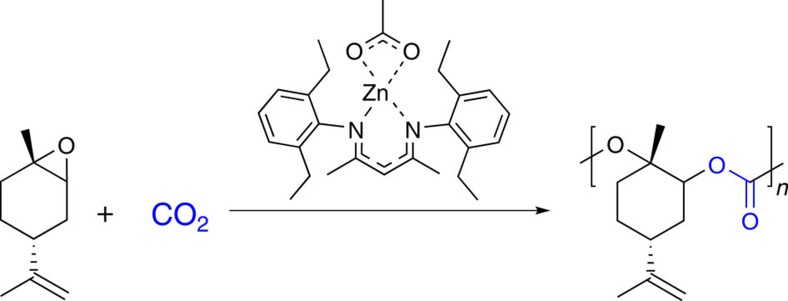
Synthetic route towards PLimC. The copolymerization of limonene oxide and CO_2_ in the presence of a *β*-diiminate zinc catalyst was discovered by Coates *et al.*^20^ and optimized in our group to yield high-molecular-weight PLimC.

**Table 1:** Glass transition temperature and tensile properties of PLimC^22^, PLimC rubber, BPA PC and silicone rubber.

**Polymer**	***T***_**g**_ **(°C)**	**Young's modulus (MPa)**	***σ***_**s**_ **(MPa)**	***ɛ*** **(%)**
PLimC	130	950	55	15
PLimC rubber	5	1.0	6.8	228
BPA PC	145	2500	65	125
Silicone rubber	−125	1.0	4.8–7.0	100–400

BPA PC, bisphenol A polycarbonate; PLimC, poly(limonene carbonate).

Data for PLimC rubber is taken from a cured sample of PLimC-B3MP with initially 2% unsaturation; for a more comprehensive table of polymers, see Supplementary Table 1.
