# Supplementary material for: Bio-based polycarbonate as synthetic toolbox
Source: Nat Commun. 2016 Jun 15;7:11862. doi: 10.1038/ncomms11862 (PMC4912624; doi:10.1038/ncomms11862)
Supplement: Supplementary Information — Supplementary Figures 1-29, Supplementary Tables 1-10, Supplementary Discussion, Supplementary Methods and Supplementary References [file ncomms11862-s1.pdf]

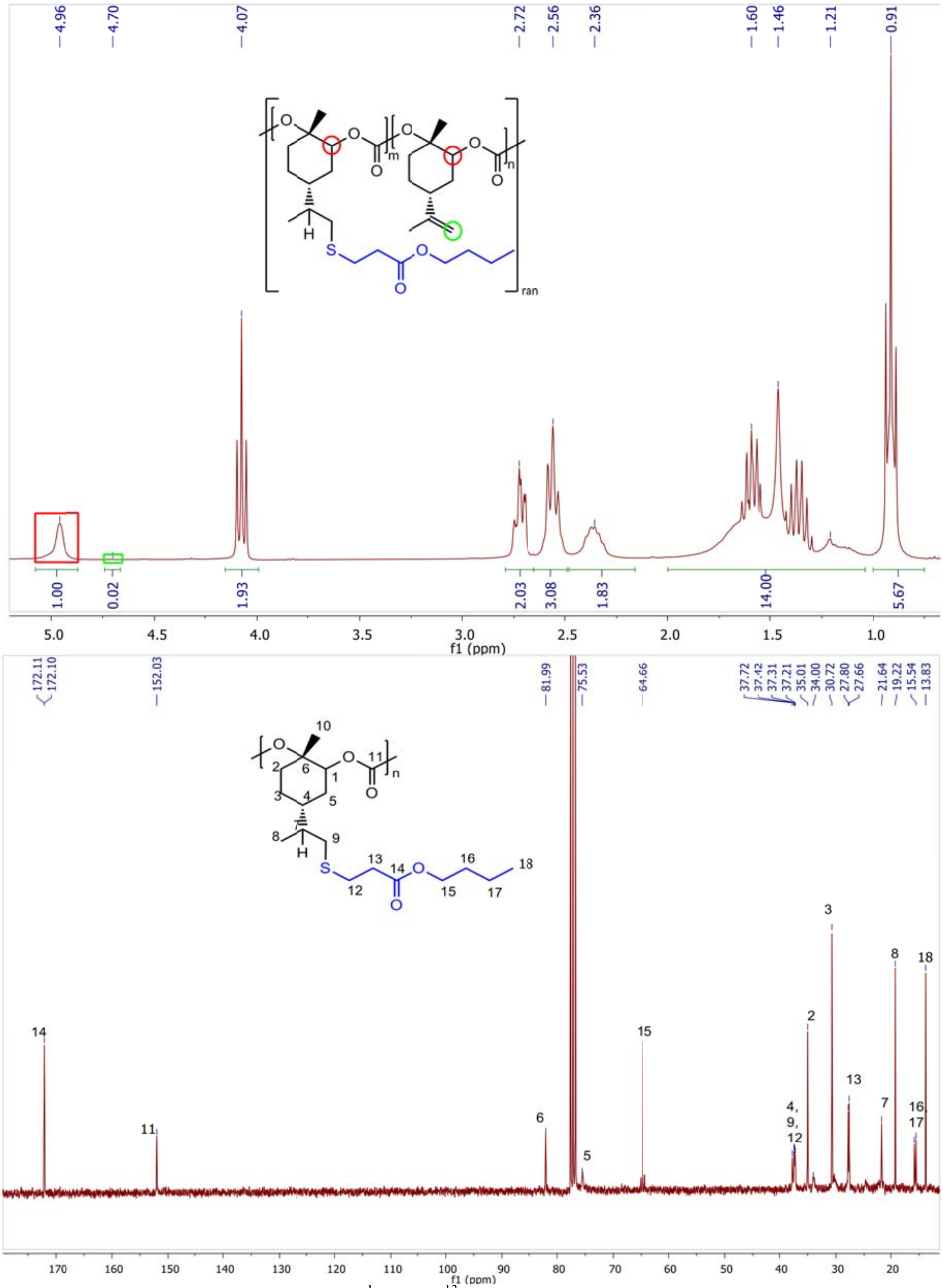

Supplementary Figure 1: Representative ^{1}H- and ^{13}C-NMR spectra (recorded in $CDCl_3$) of PLimC-B3MP$_{1.0}$ after reacting PLimC for 80 min with butyl-3-mercpatopropionate to give 99% conversion. The red rectangle indicates the methine resonance (adjacent to carbonate group) of both the unfunctionalized and the functionalized repeating unit, whereas the green rectangle marks the region where the protons of the (unfunctionalized) double bond are resonating. Since unsaturation is very low in all samples the region 5.2 – 4.6 ppm is magnified for all PLimC-B3MP samples in Supplementary Figure 2.

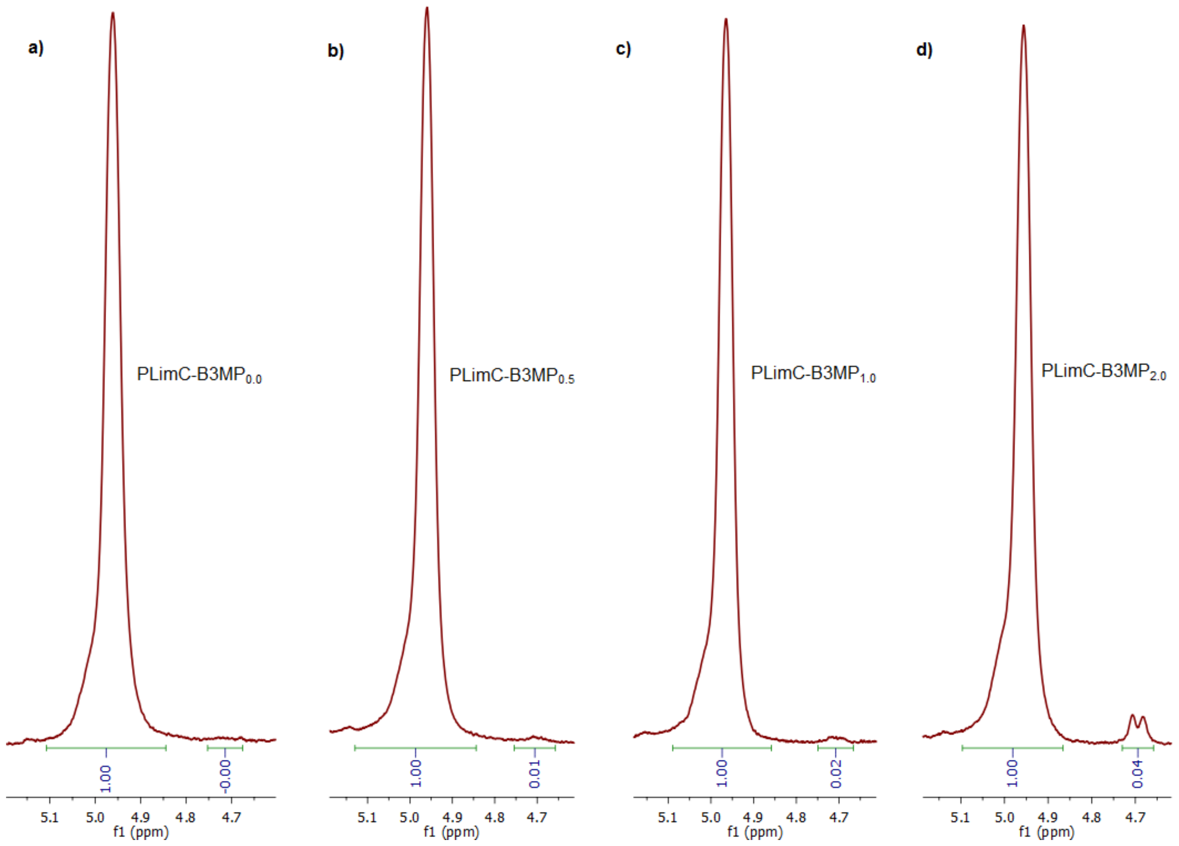

Supplementary Figure 2: Magnification of the methine and double bond proton resonances in the ^{1}H-NMR spectra of PLimC-B3MP with a) 0%, b) 0.5%, c) 1.0% and d) 2% unsaturation in the backbone. The degree of unsaturation has been determined by integration of the signal at 4.7 ppm with respect to the methine proton at 5.0 ppm.

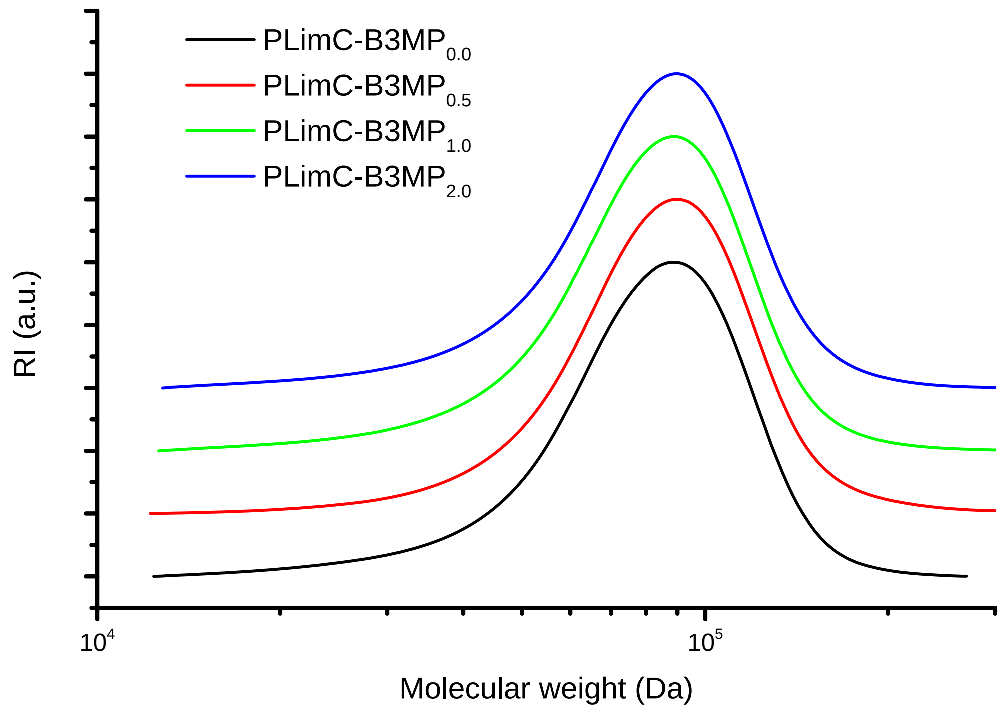

Supplementary Figure 3: Molecular weight distribution of the PLimC-B3MP samples with different DFs. Measurements were performed on a GPC with chloroform as eluent.

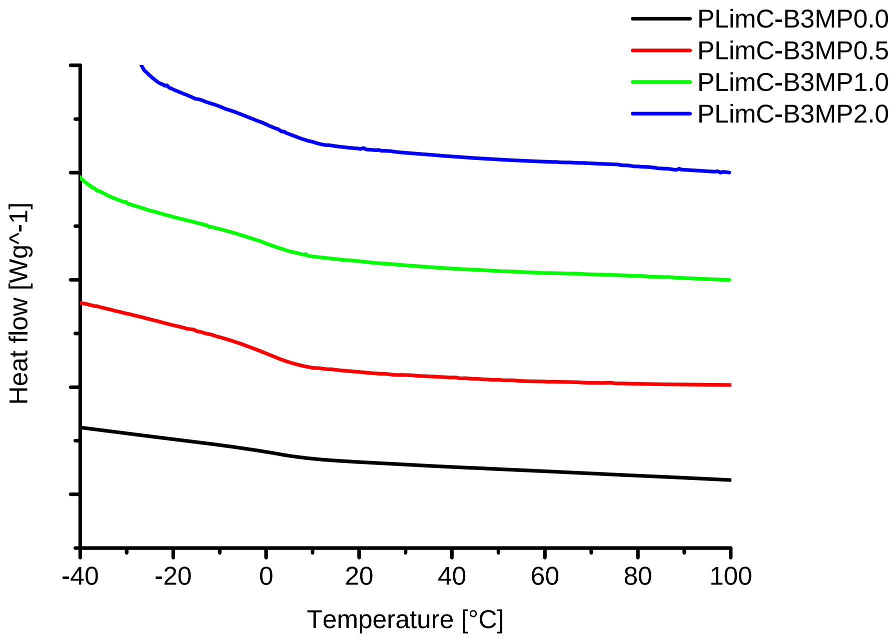

Supplementary Figure 4: Second heating cycle in DSC experiment of PLimC B3MP samples with various DFs recorded at 10 K min^{-1} in N_2 atmosphere. All samples exhibit a glass transition at about 5 °C, as it was expected, since all samples exhibit a DF close to 100%.

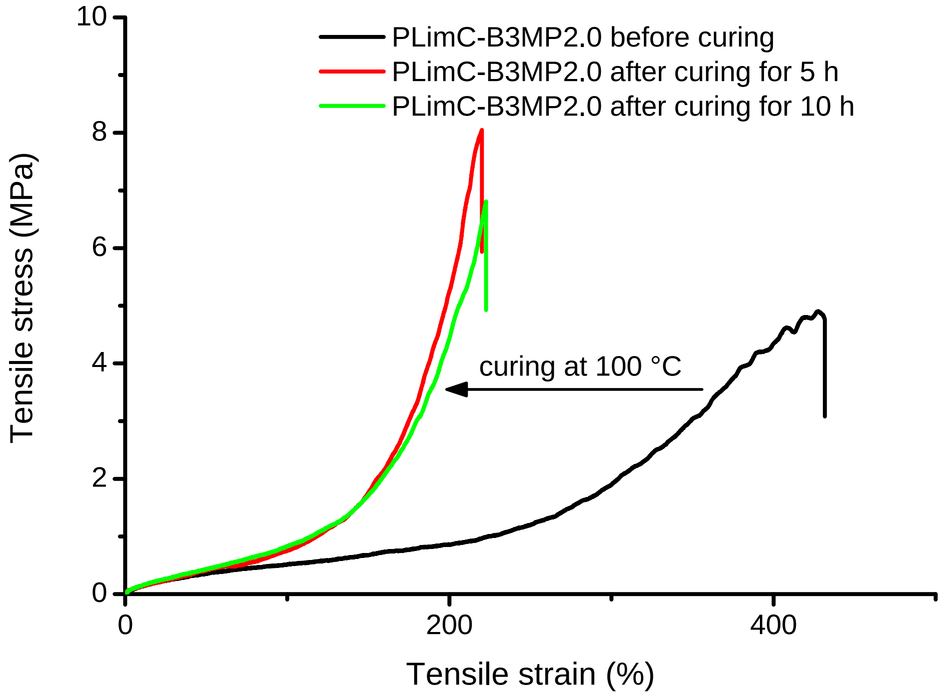

Supplementary Figure 5: Effect of curing (time) on the tensile properties of PLimC-B3MP$_{2.0}$. The diagram shows the tensile tests on specimens of PLimC-B3MP$_{2.0}$ performed before curing (black) and after curing in air at 100 °C for 5 h (red) or 10 h (green), respectively. While the curing for 5 h has a dramatic effect on the tensile properties compared to the uncured sample, longer curing time (in total 10 h at 100 °C) does not change the tensile properties any further.

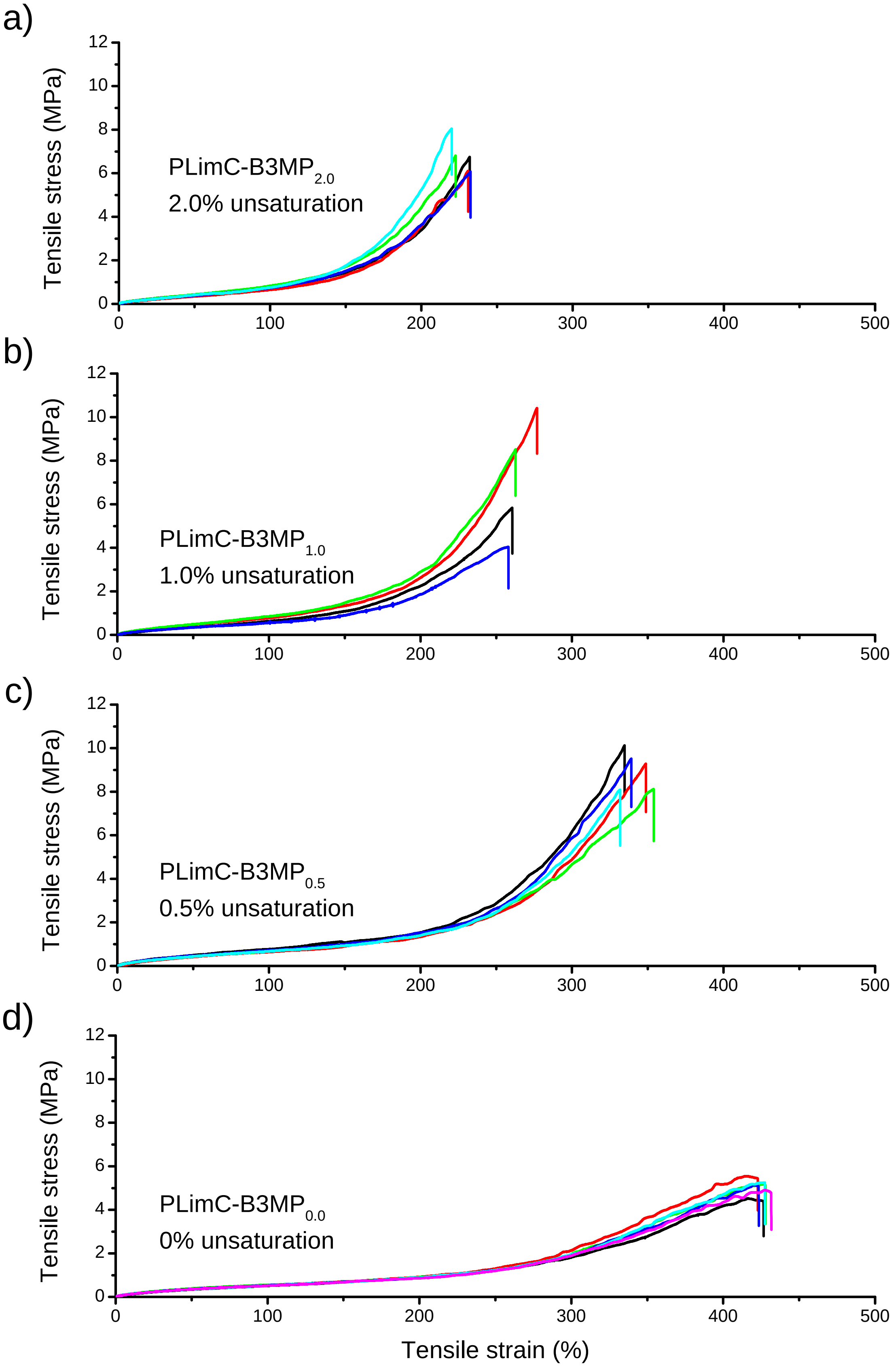

Supplementary Figure 6: Stress-strain diagram of tensile testing of cured PLimC-B3MP specimens with a) 2.0%, b) 1.0%, c) 0.5% and d) 0.0% unsaturation in the (uncured) sample. The measurements were performed at a speed of 5 mm min^{-1} on specimen with a width of 2 mm, a length of 20 mm and a thickness of 150 µm. The films were cast from a methylene chloride solution, dried *in vacuo* at 20 °C and subsequently cured at 100 °C for 5 h in air.

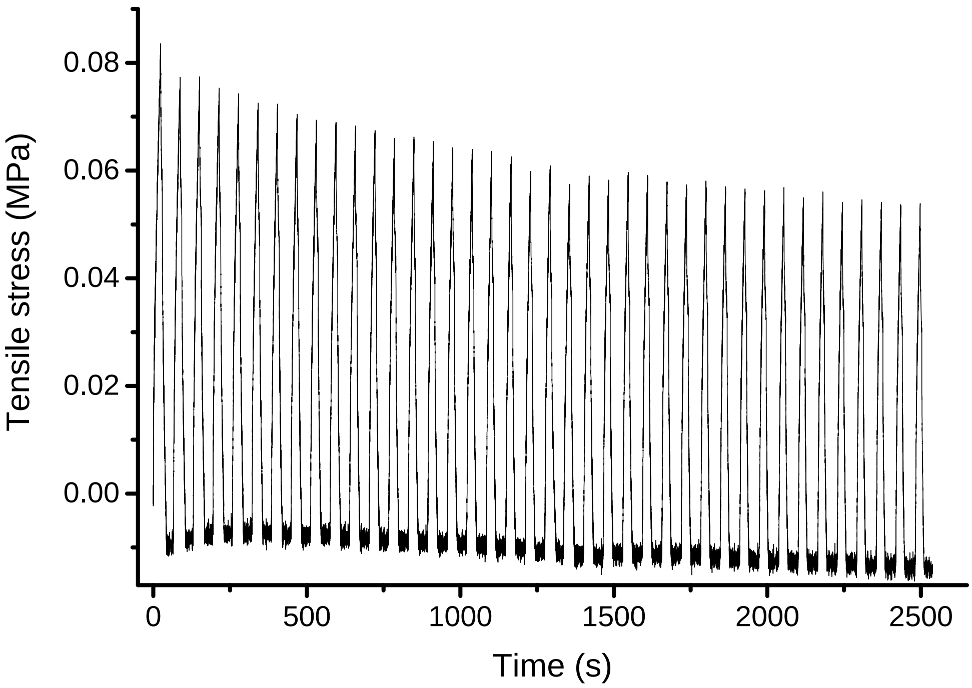

Supplementary Figure 7: Evaluation of the elasticity of a cured specimen of PLimC-B3MP$_{2.0}$ by cyclic elongation to 20% strain (40 cycles, 5 s strain, 10 s relaxation, strain rate 10 mm min^{-1}). The strain is not completely reversible (maximum stress drops from 0.08 MPa for each consecutive cycle down to 0.06 MPa for the last cycle), as the T_g (5 °C) of the polymer is very close to the testing temperature of 21 °C, i.e. the slow dynamics can be assigned to the slow segmental motion of the polymer backbone. To improve reversibility in the strain experiment, the modification of PLimC with thiols that lower the T_g well below 0 °C should be used.

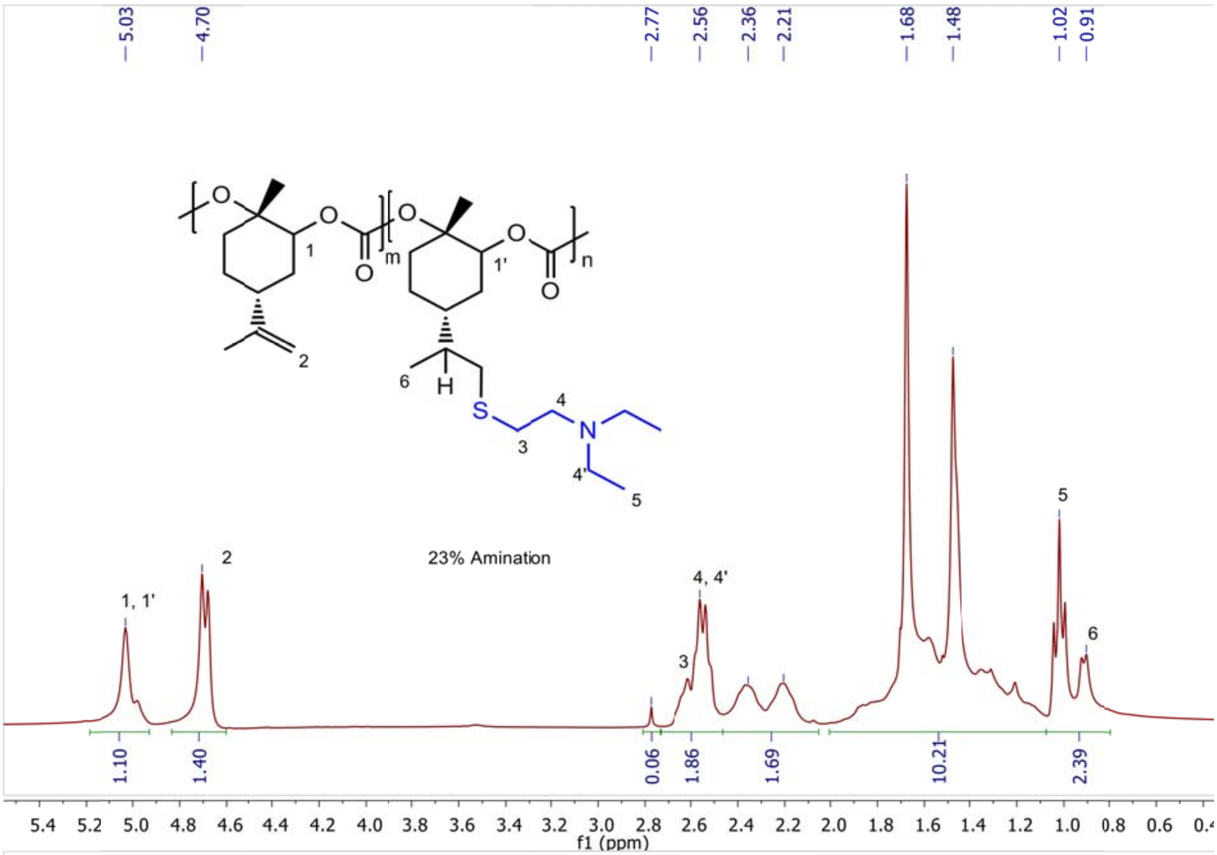

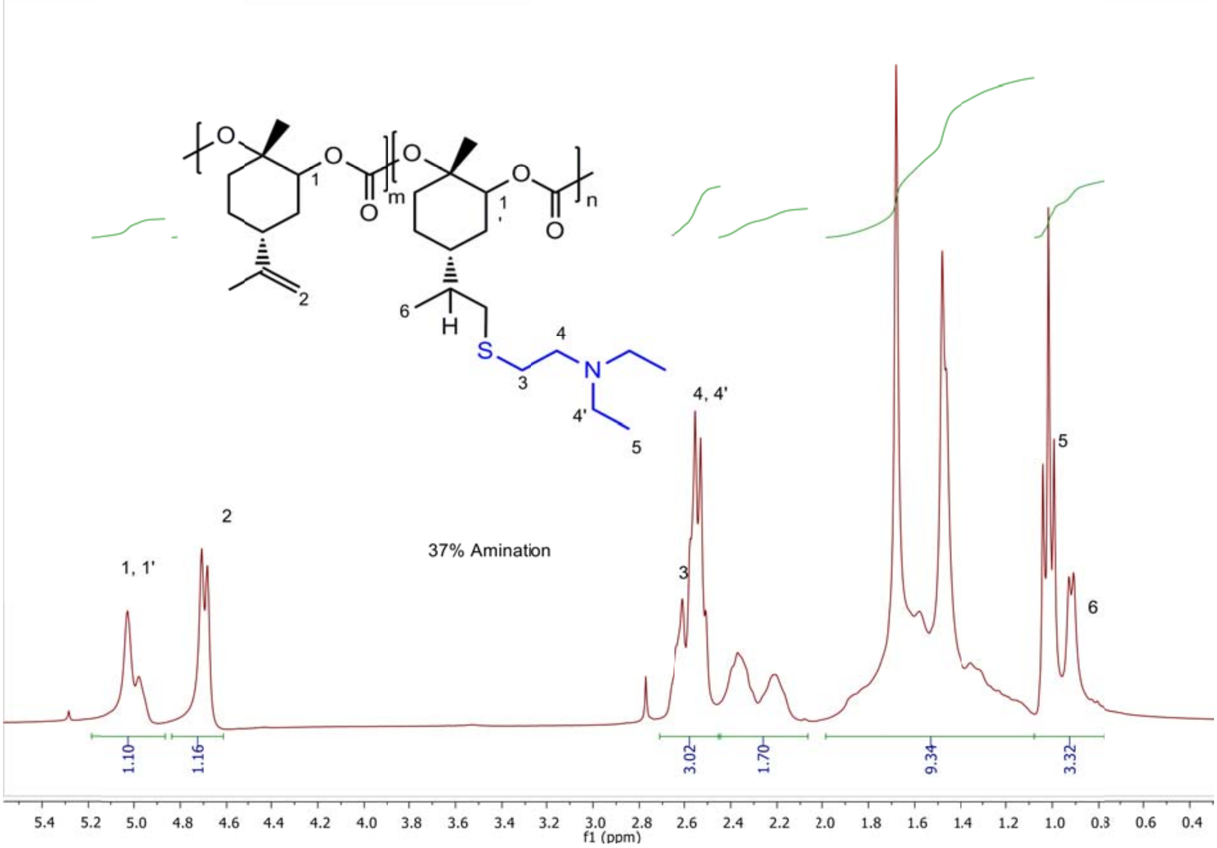

Supplementary Figure 8: ^{1}H-NMR spectrum (recorded in $CDCl_3$) of PLimC-N23 (57 kDa) after reacting PLimC (treated with antioxidant before) for 3 h with 2-(diethylamino)ethanethiol hydrochloride to give a degree of functionalization of 23%. ^{1}H-NMR spectrum (recorded in $CDCl_3$) of PLimC-N37 (85 kDa) after reacting PLimC for 3 h with 2-(diethylamino)ethanethiol hydrochloride to give a degree of functionalization of 37%.

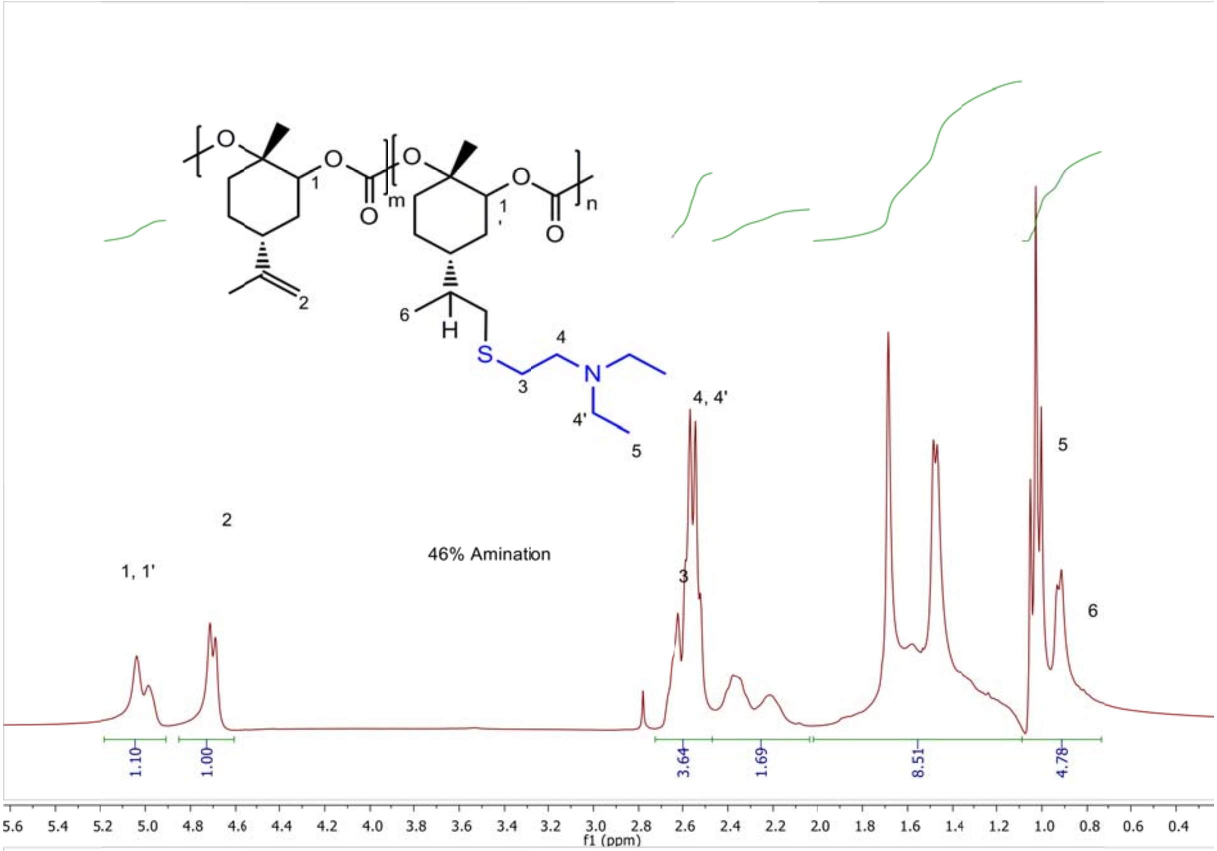

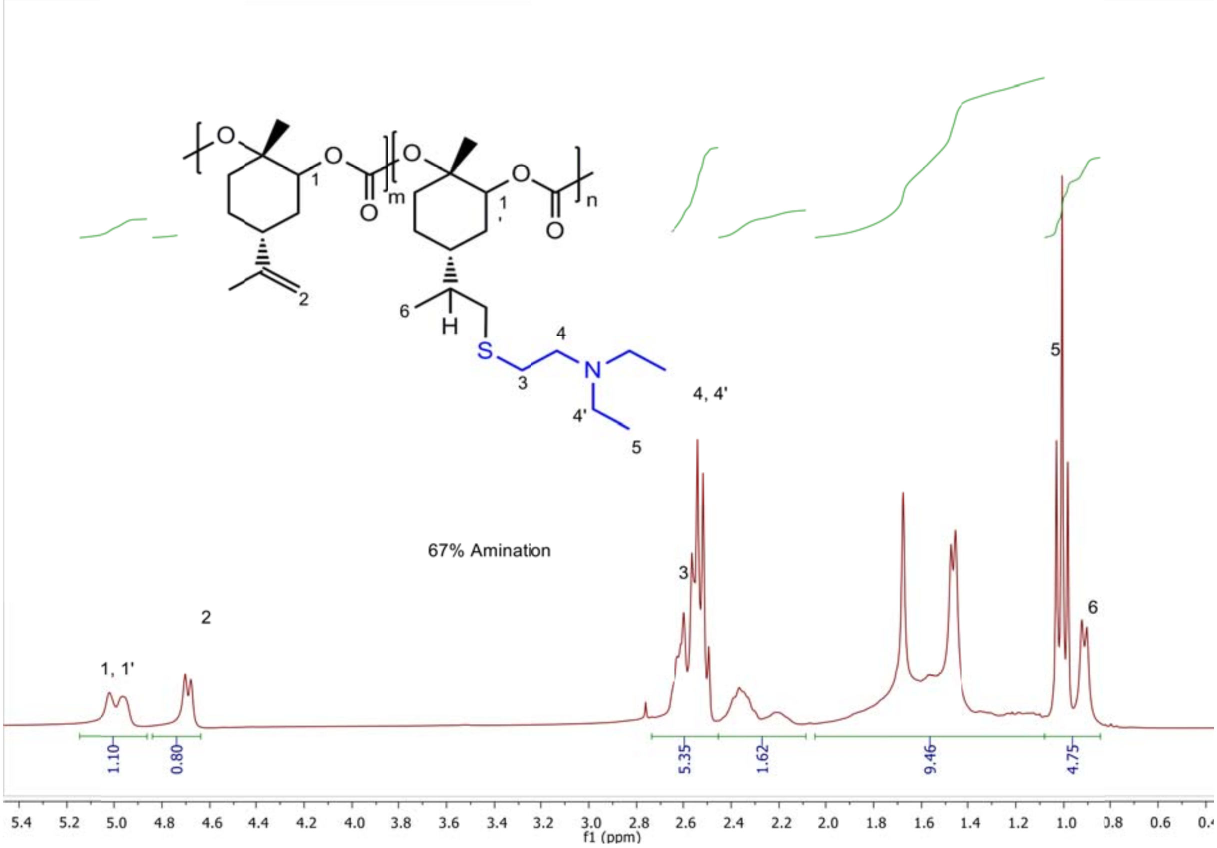

Supplementary Figure 9: ^{1}H-NMR spectra (recorded in $CDCl_3$) of PLimC-N46 and PLimC-N67 (both 85 kDa) after reacting PLimC for 4 h or 6 h with 2-(diethylamino)ethanethiol hydrochloride to give a degree of functionalization of 46% and 67%, respectively.

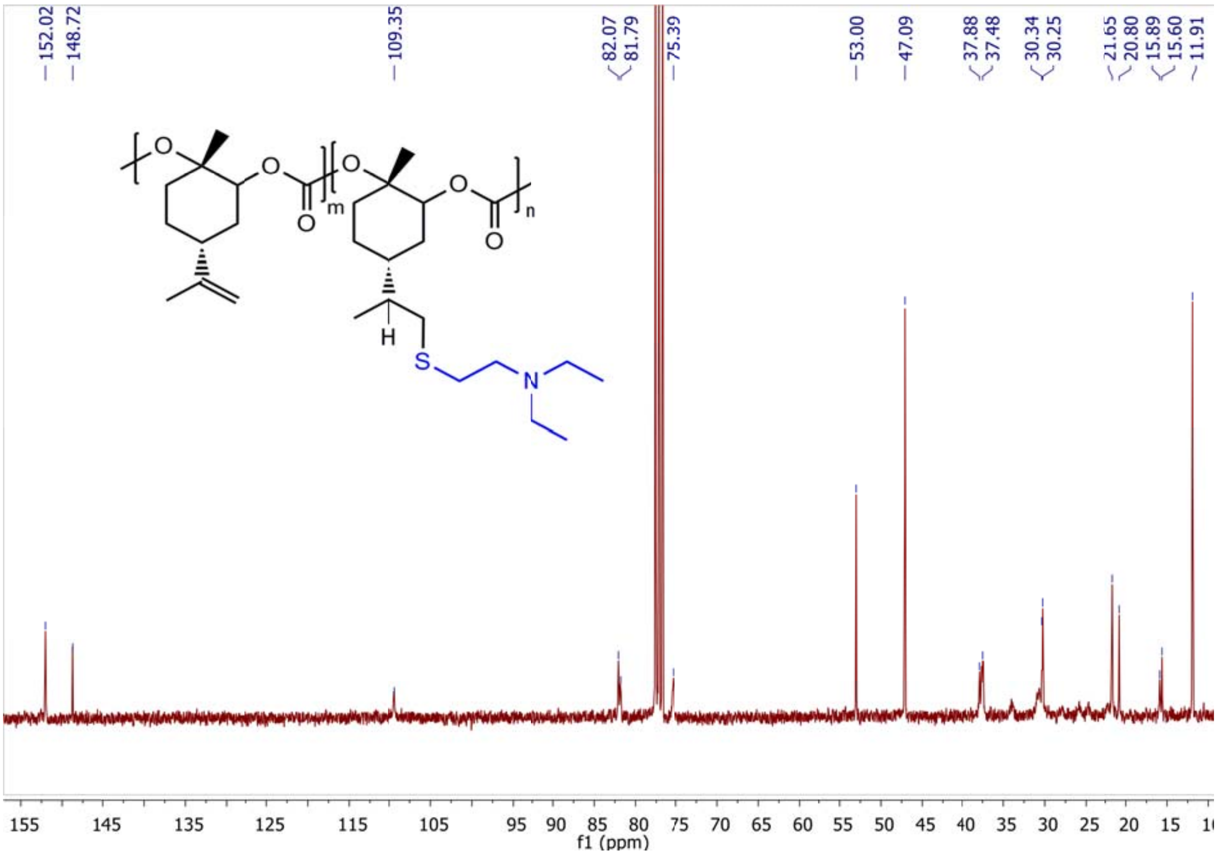

Supplementary Figure 10: Representative ^{13}C-NMR spectrum (recorded in $CDCl_3$) of PLimC-N67 (85 kDa) after reacting PLimC for 6 h with 2-(diethylamino)ethanethiol hydrochloride to give a degree of functionalization of 67%.

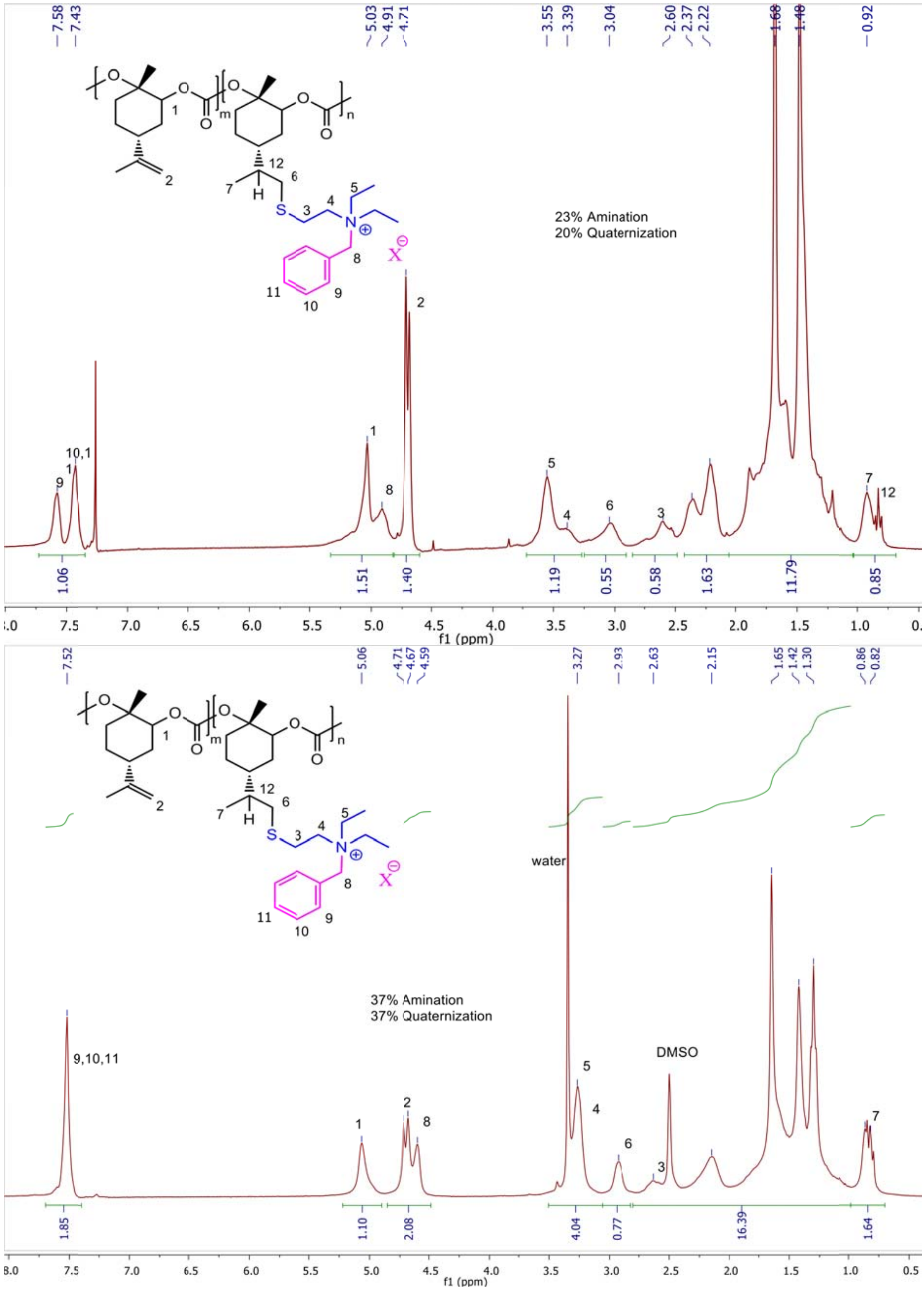

Supplementary Figure 11: ^{1}H-NMR spectrum (recorded in $CDCl_3$) of PLimC-NQ20 (57 kDa) after reacting PLimC-23N for 24 h with benzyl bromide to give nearly quantitative quaternization of the amine. ^{1}H-NMR spectrum (recorded in DMSO-d_6) of PLimC-NQ37 (85 kDa) after reacting PLimC-N37 for 24 h with benzyl bromide to give quantitative quaternization of the amine.

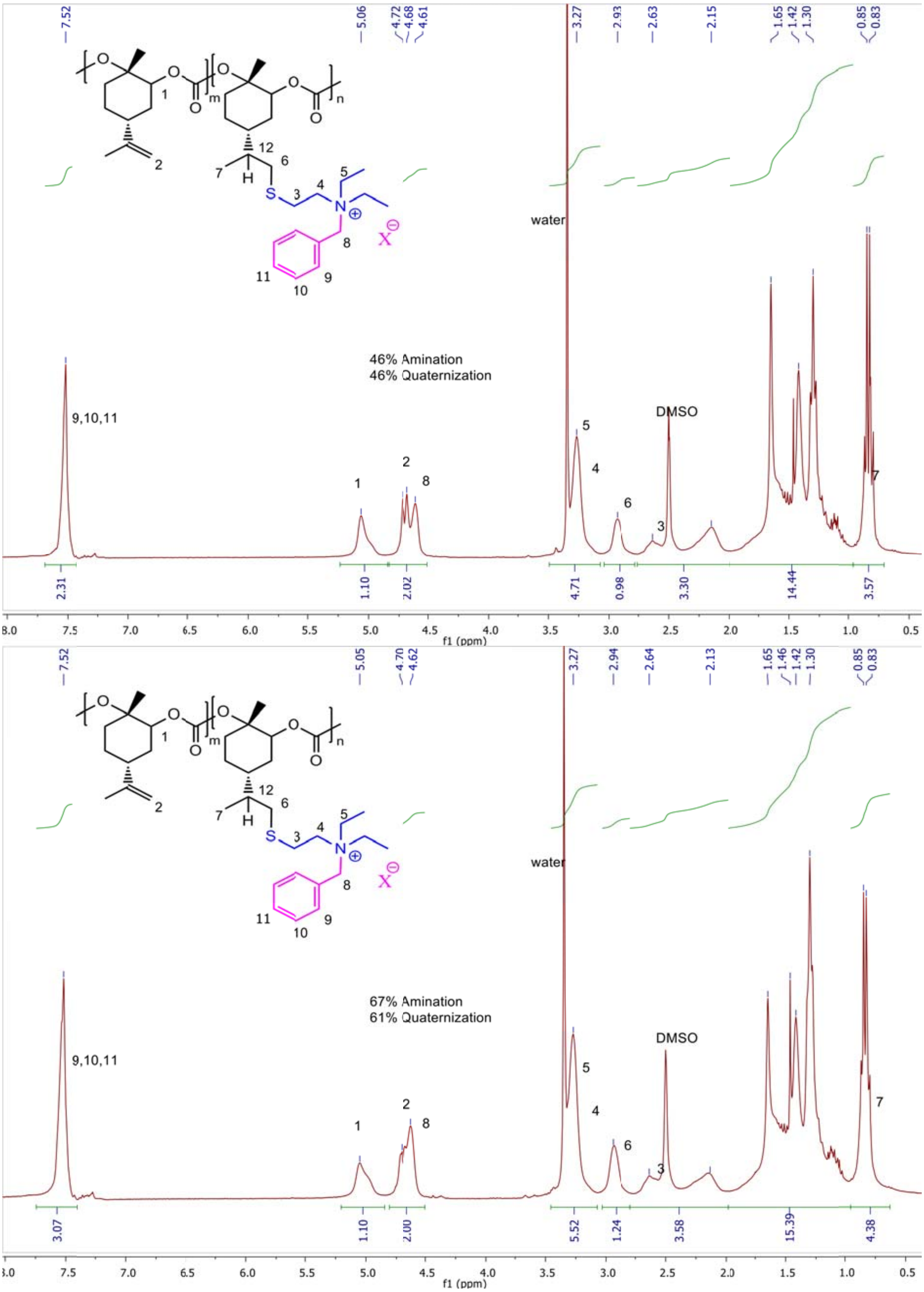

Supplementary Figure 12: ^{1}H-NMR spectra (recorded in DMSO-d_6) of PLimC-NQ46 and PLimC-NQ61 (both 85 kDa) after reacting PLimC-N46 and PLimC-N67 for 24 h with benzyl bromide to give quantitative or 90% quaternization, respectively.

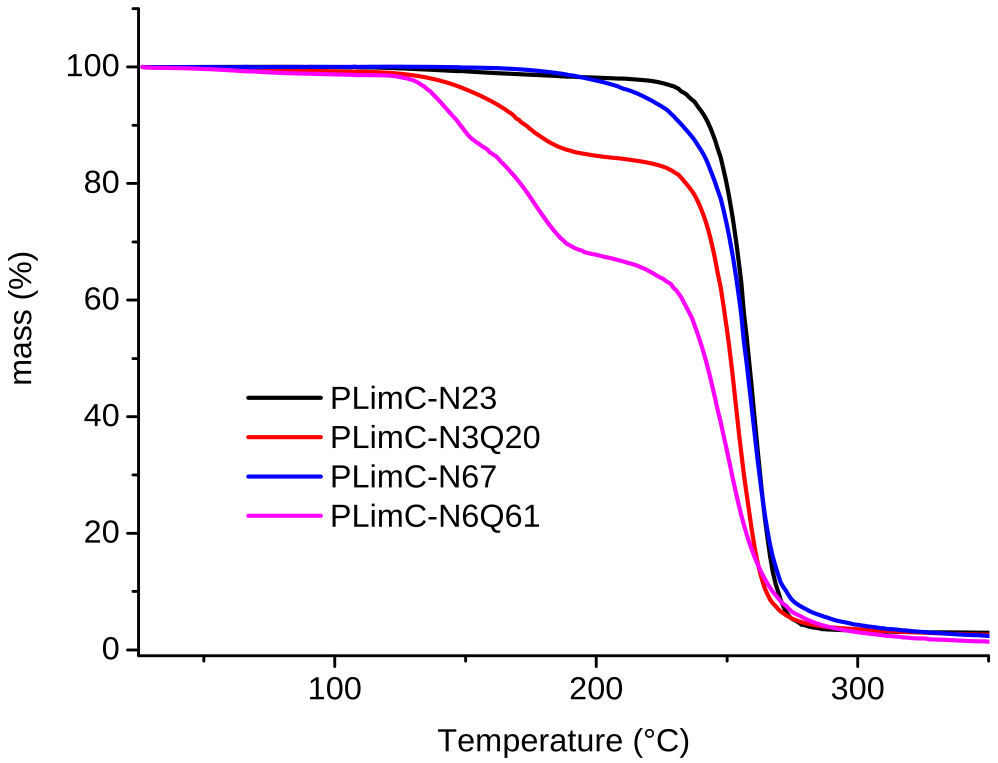

Supplementary Figure 13: Thermogravimetric analysis of aminated (PLimC-N) and quaternized (PLimC-NQ) samples of PLimC with different DF, measured at 10 K min^{-1} under N_2 atmosphere.

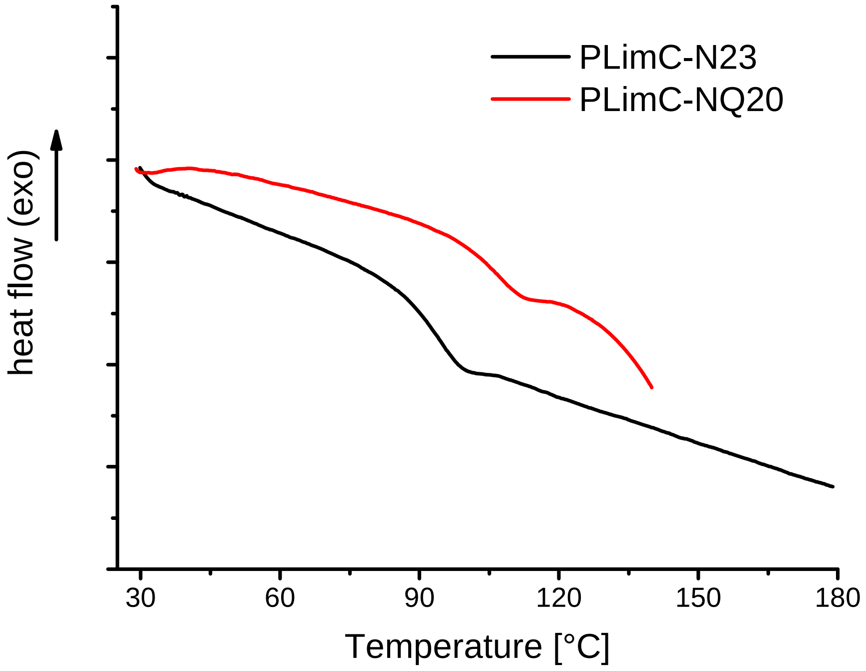

Supplementary Figure 14: Second heating runs of DSC experiment of aminated (PLimC-N23) and quaternized (PLimC-NQ20) samples, measured at 10 K min^{-1} in N_2 atmosphere. The trace of PLimC-NQ20 is cut off at 140 °C, since decomposition of the sample is already in full progress.

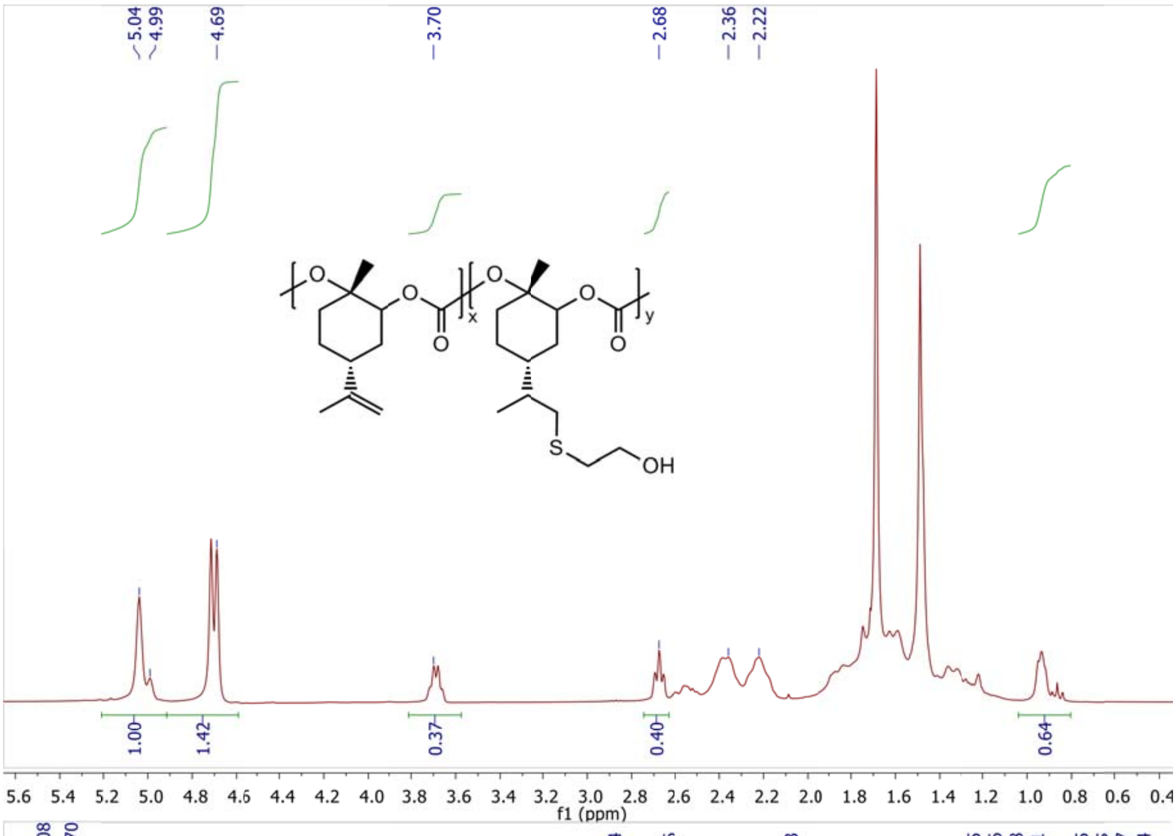

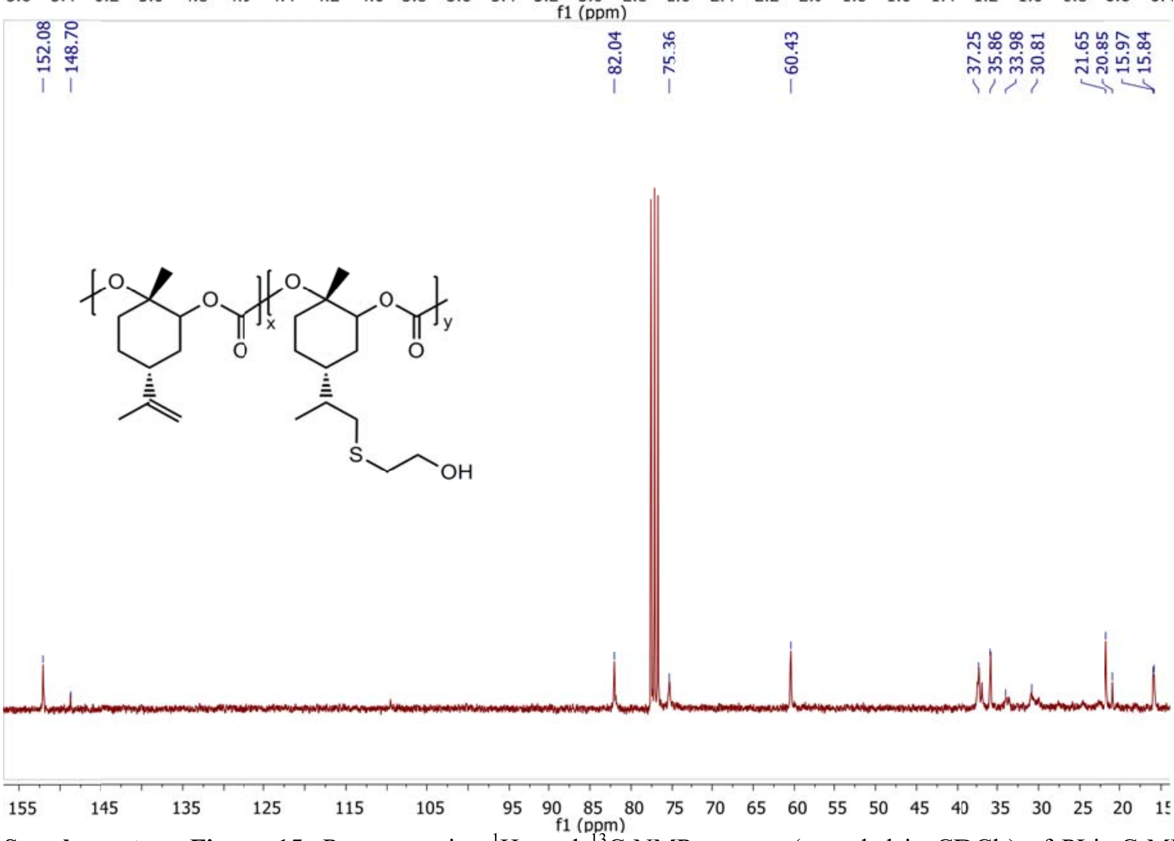

Supplementary Figure 15: Representative ^{1}H- and ^{13}C-NMR spectra (recorded in $CDCl_3$) of PLimC-ME18 after reacting PLimC for 1 h with mercaptoethanol to give 18% conversion.

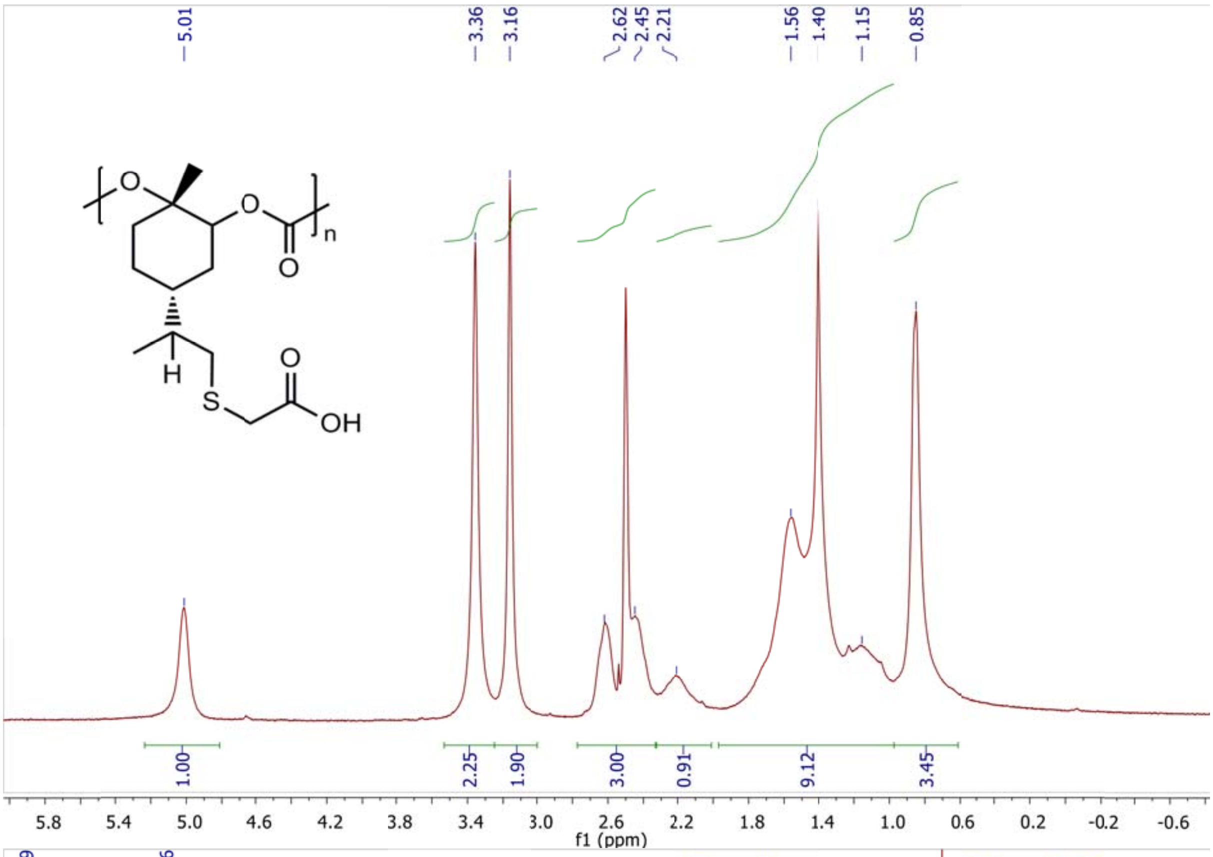

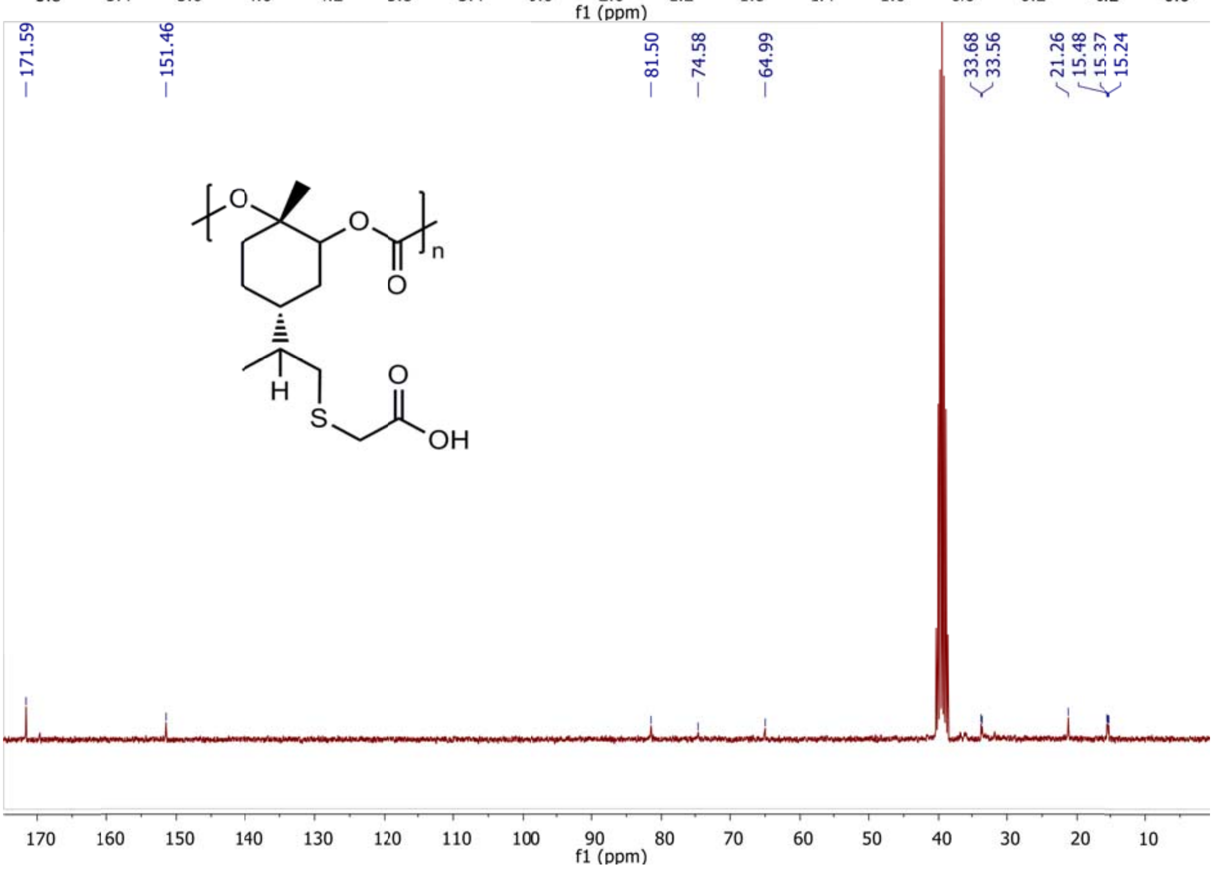

Supplementary Figure 16: ^{1}H- and ^{13}C-NMR spectra (recorded in DMSO-d^{6}) of PLimC-MAc after reacting PLimC for 3 h with mercaptoacetic acid to give quantitative conversion.

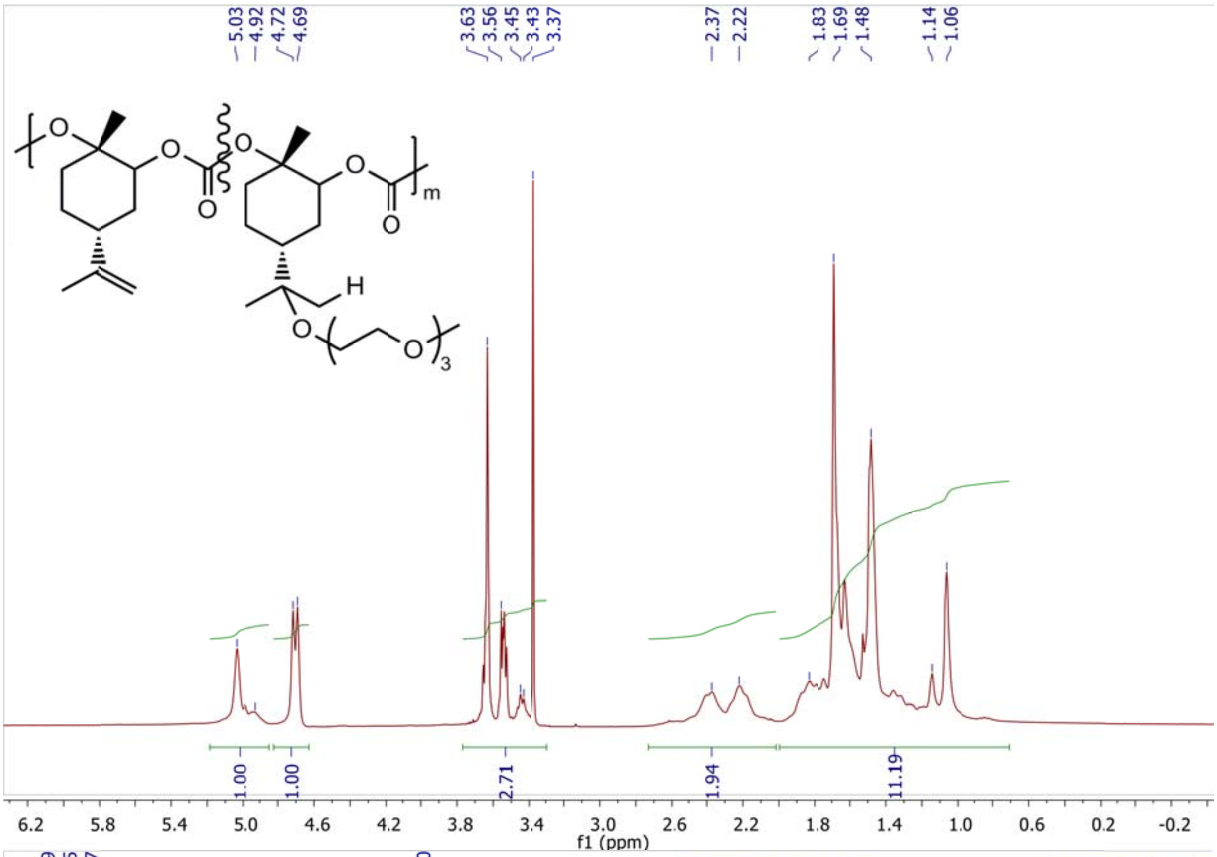

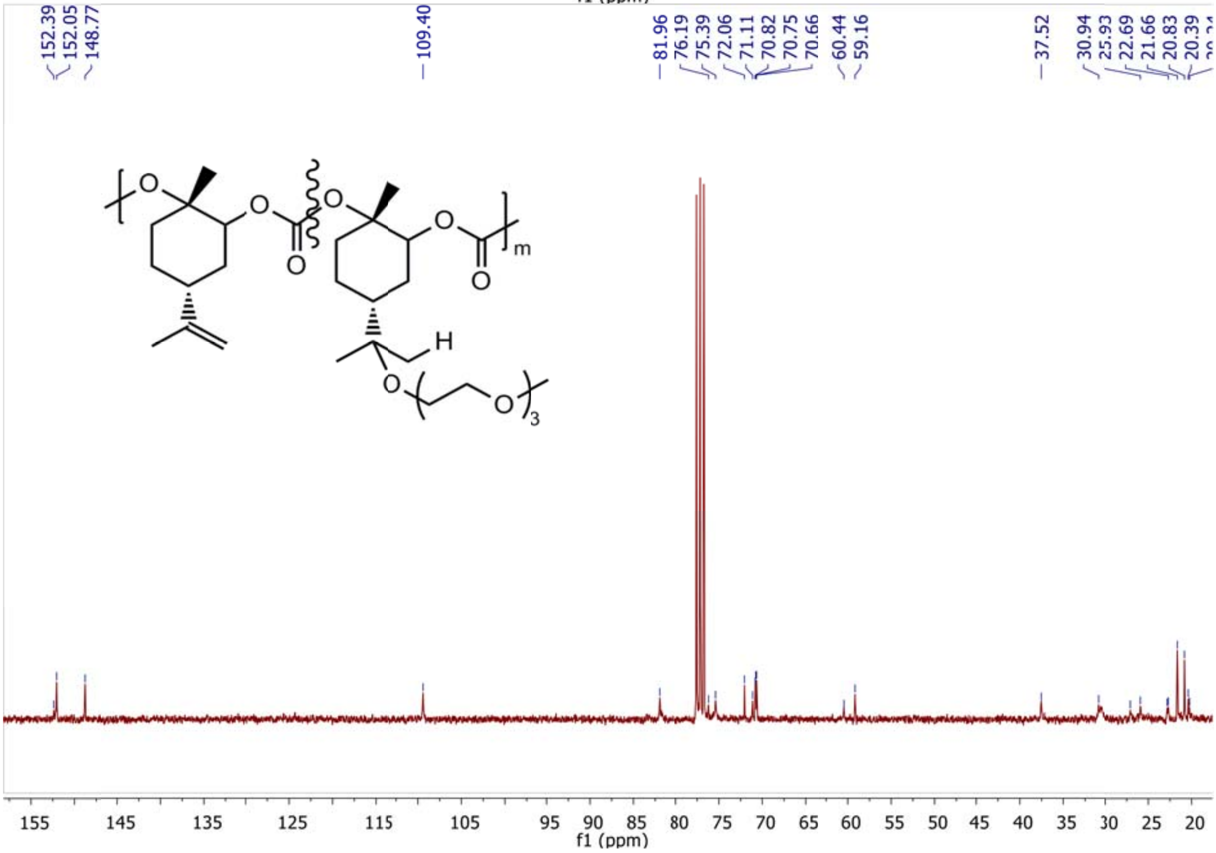

Supplementary Figure 17: ^{1}H- and ^{13}C-NMR spectra (recorded in $CDCl_3$) of PLimC-PEG after reacting PLimC for 68 h with PEG-3-OH to give a degree of functionalization of 18%.

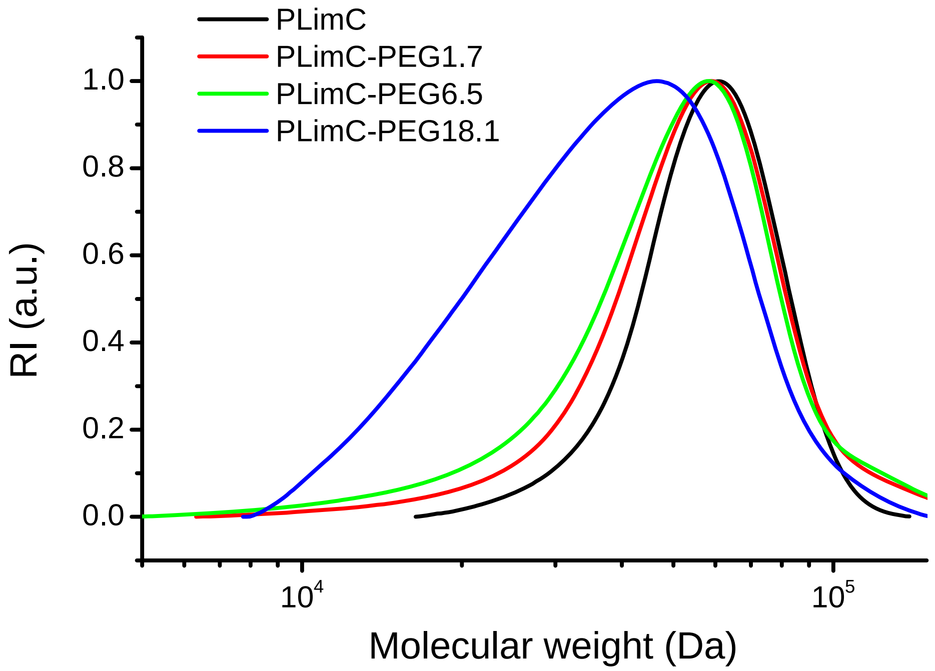

Supplementary Figure 18: GPC molar mass distribution (eluent was chloroform, calibration was performed with PS standards) of PLimC and the PEG functionalized samples, showing the effect of the acidic conditions, that degrade PLimC after prolonged exposure.

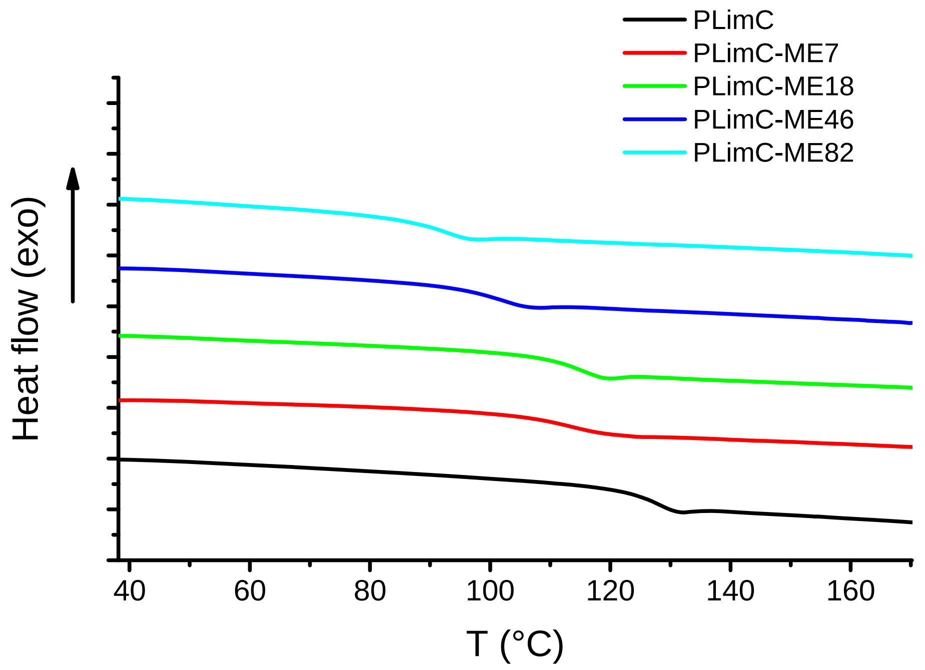

Supplementary Figure 19: Development of T_g with increasing functionalization of PLimC with ME. For longer reaction times and higher degree of functionalization the T_g is rising again, because of intermolecular cross-linking between the double bonds.

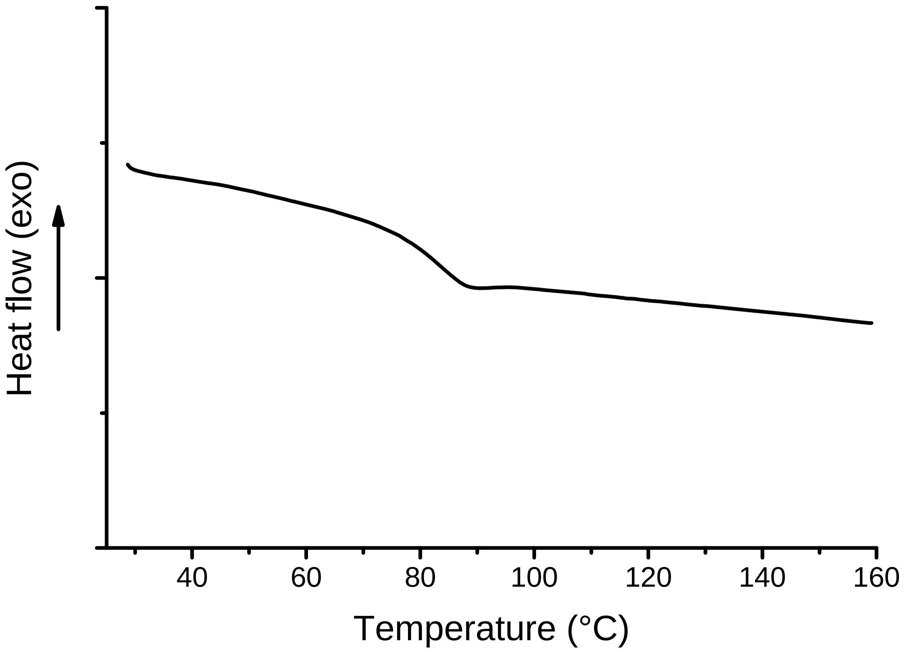

Supplementary Figure 20: DSC thermogram of PLimC-MAc measured at 10 K min^{-1} in N_2 atmosphere showing a glass transition of the sample at 82 °C.

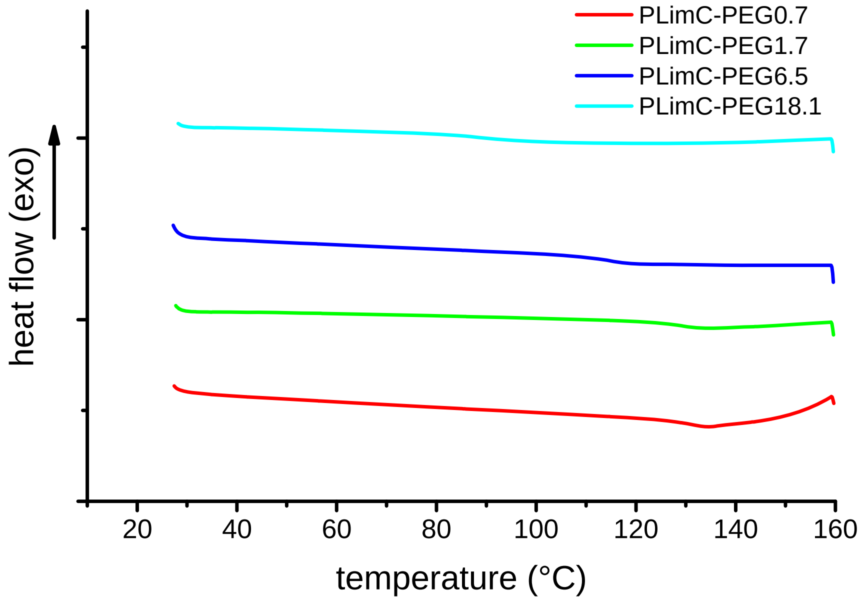

Supplementary Figure 21: Second run of DSC experiments of PLimC functionalized with various amounts of PEG-3-OH by acid-catalyzed electrophilic addition of PEG-3-OH.

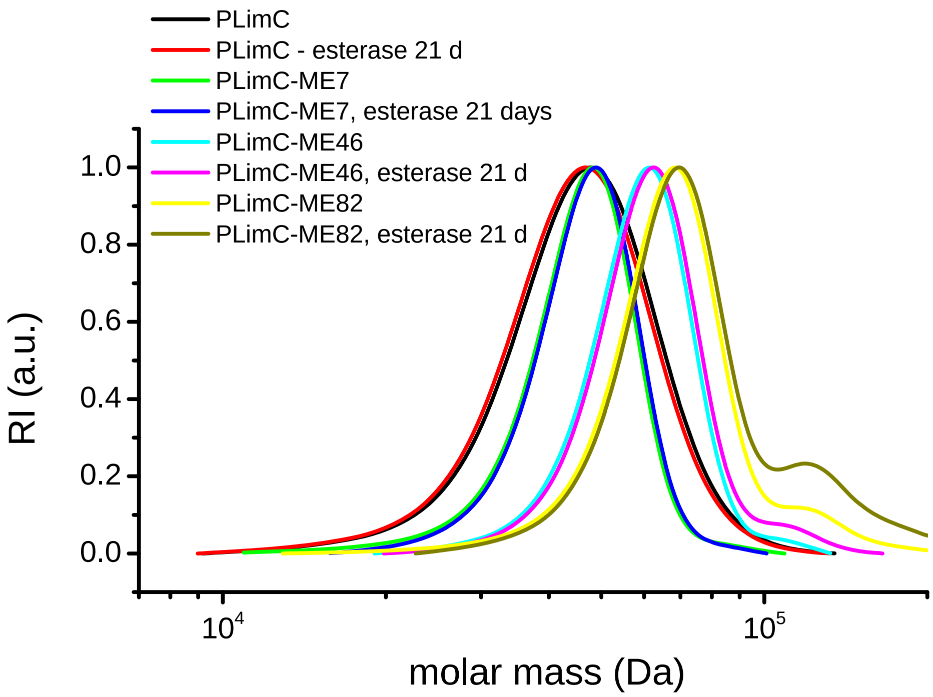

Supplementary Figure 22: Molar mass distribution of PLimC, PLimC-ME7/46/82 films before and after immersion for 21 days in an esterase buffer (pH 9) suspension. For all samples no significant change of molar mass distribution is observed, suggesting stability of the samples under these conditions. The eluent for GPC measurements was chloroform.

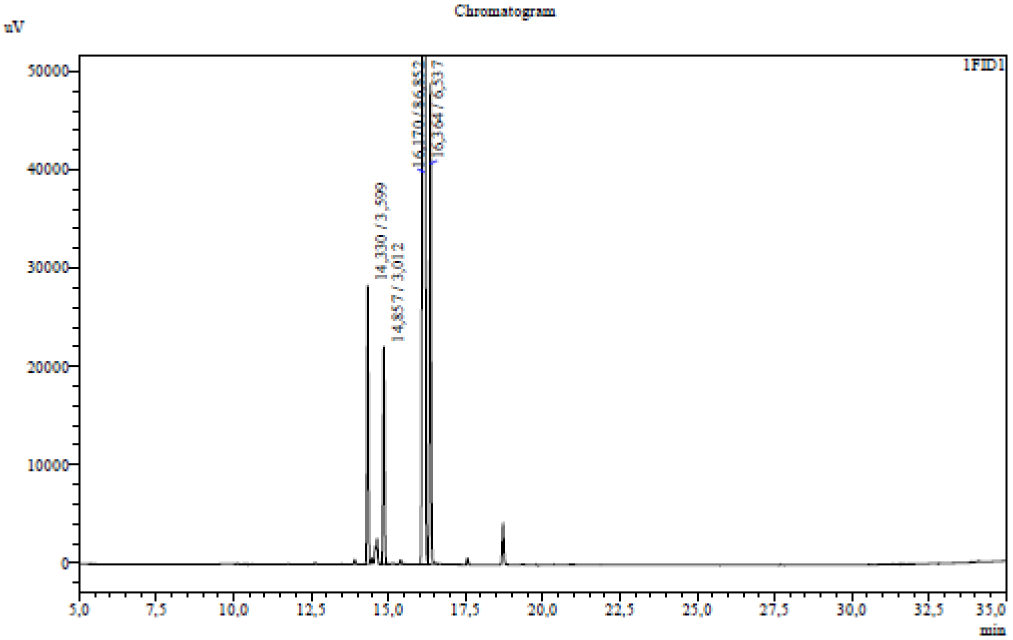

Peak Table

FID1

Peak#	Ret. Time	Area%	Area	Height
1	14,330	3,599	88836	28335
2	14,857	3,012	74344	22143
3	16,170	86,852	2143677	556919
4	16,364	6,537	161346	48906
Total		100,000	2468203	656304

Supplementary Figure 23: GC analysis of menth-1-ene with a retention time of 16.4 min, after filtration step to remove the catalyst.

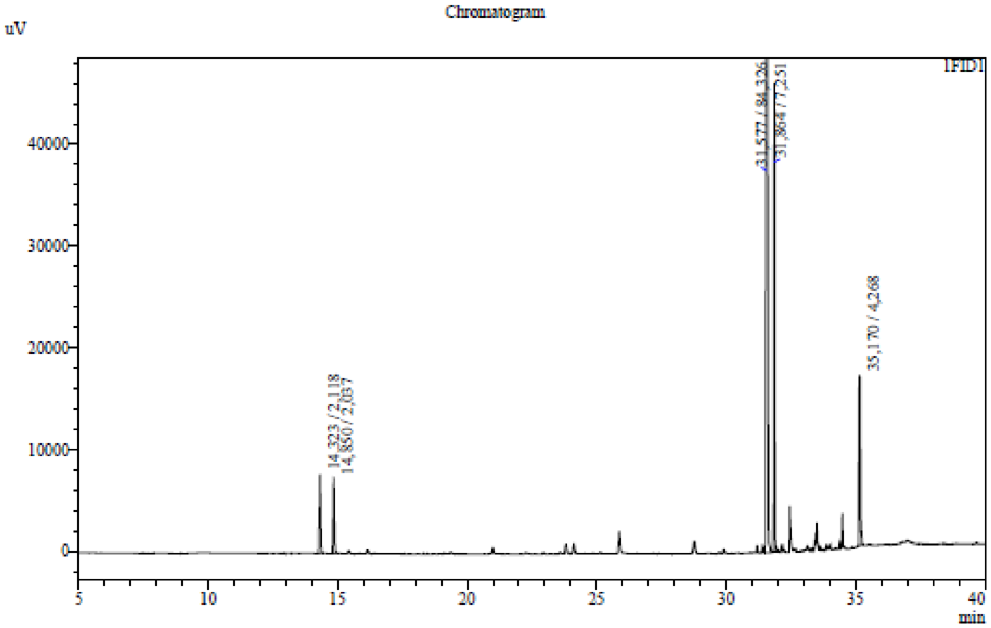

Peak Table

FID1

Peak#	Ret. Time	Area%	Area	Height
1	14,323	2,118	25801	7766
2	14,850	2,037	24812	7379
3	31,577	84,326	1027055	430913
4	31,864	7,251	88309	45974
5	35,170	4,268	51984	16696
Total		100,000	1217961	508727

Supplementary Figure 24: GC analysis of the bromohydrin of menth-1-ene (MenBrOH) with retention times of 31.6 (*cis*) and 31.9 (*trans*) min, which was directly converted into the epoxide without prior purification.

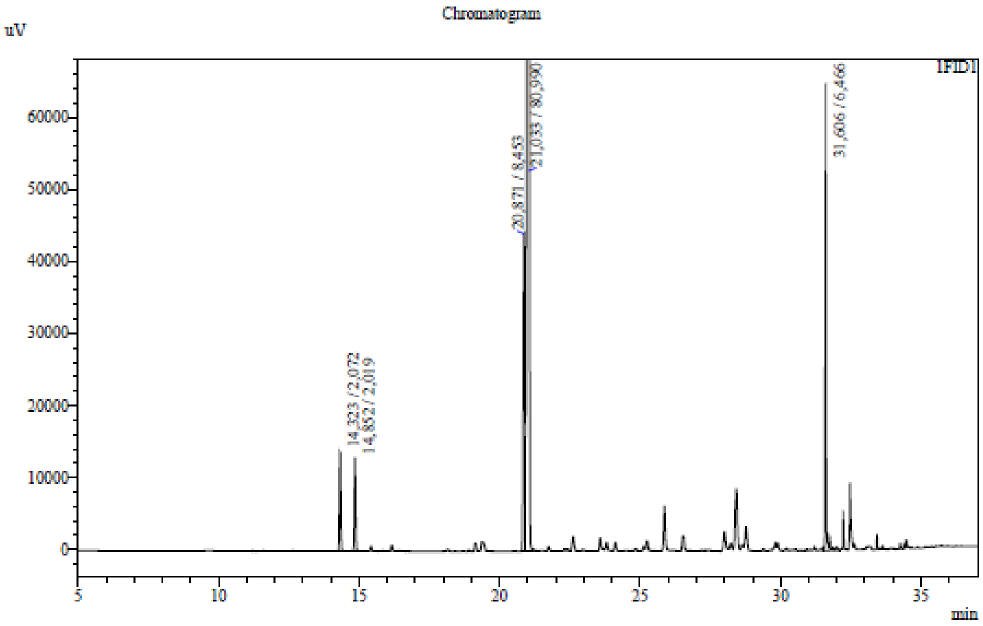

Peak Table

FID1

Peak#	Ret. Time	Area%	Area	Height
1	14.323	2.072	45127	13779
2	14.852	2.019	43973	12986
3	20.871	8.453	184075	44005
4	21.033	80.990	1763692	415694
5	31.606	6.466	140807	64610
Total		100.000	2177674	551074

Supplementary Figure 25: GC analysis of the MenO with retention times of 20.8 (*cis*) and 21.0 (*trans*) min, which was afterwards treated with NaH/ MeI and distilled. In the peak table below, the excess of *trans*-isomer is proven by the relative areas, i.e. 81% for *trans*-MenO.

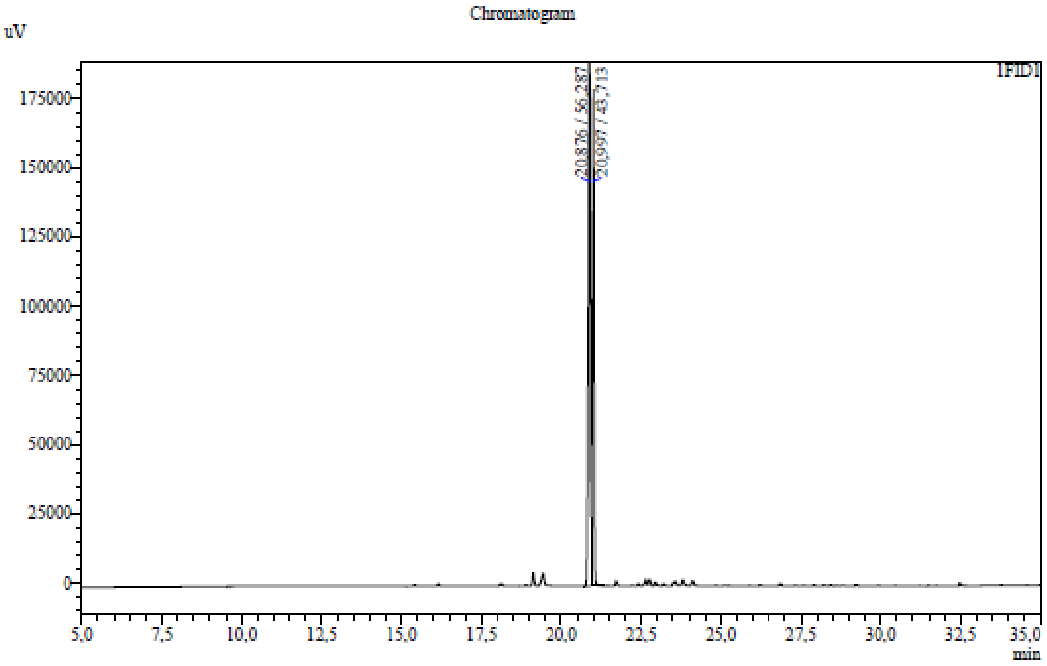

Peak Table

FID1

Peak#	Ret. Time	Area%	Area	Height
1	20,876	56,287	855222	208189
2	20,997	43,713	664184	179247
Total		100,000	1519406	387436

Supplementary Figure 26: GC analysis of the precipitation bath of PMenC showing mainly *cis*-MenO (20.9 min) and *trans*-MenO (21.0 min). In the peak table below, the accumulation of *cis*-isomer is proven by the relative areas, i.e. 56% for *cis*-MenO compared to 44% *trans*-MenO, indicating the preferential incorporation of the latter into PMenC.

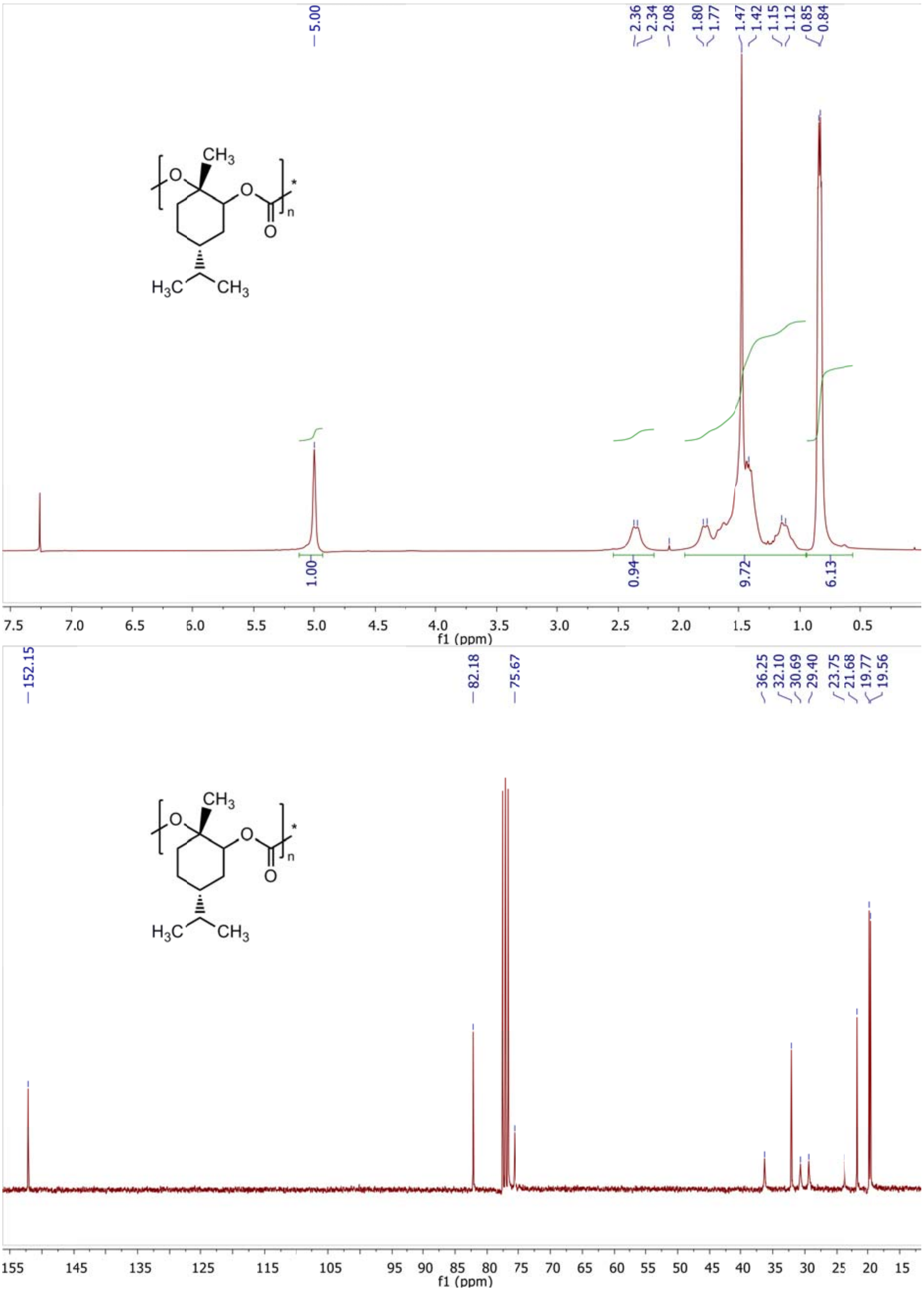

Supplementary Figure 27: ^{1}H- and ^{13}C-NMR spectra (recorded in $CDCl_3$) of PMenC, which was produced by copolymerisation of *trans*-menthene oxide with CO_2 in the presence of a Zn catalyst. The preferential incorporation is also represented in the NMR spectra of PMenC. The ^{1}H-NMR spectrum shows a single peak at 5.00 ppm without any downfield shoulder, which would be an indication of incorporation of the *cis*-isomer into the backbone. This argument is further supported by the ^{13}C-NMR spectrum of PMenC, which shows only one carbonyl resonance at 152.2 ppm i.e. no stereo-irregularities are present.

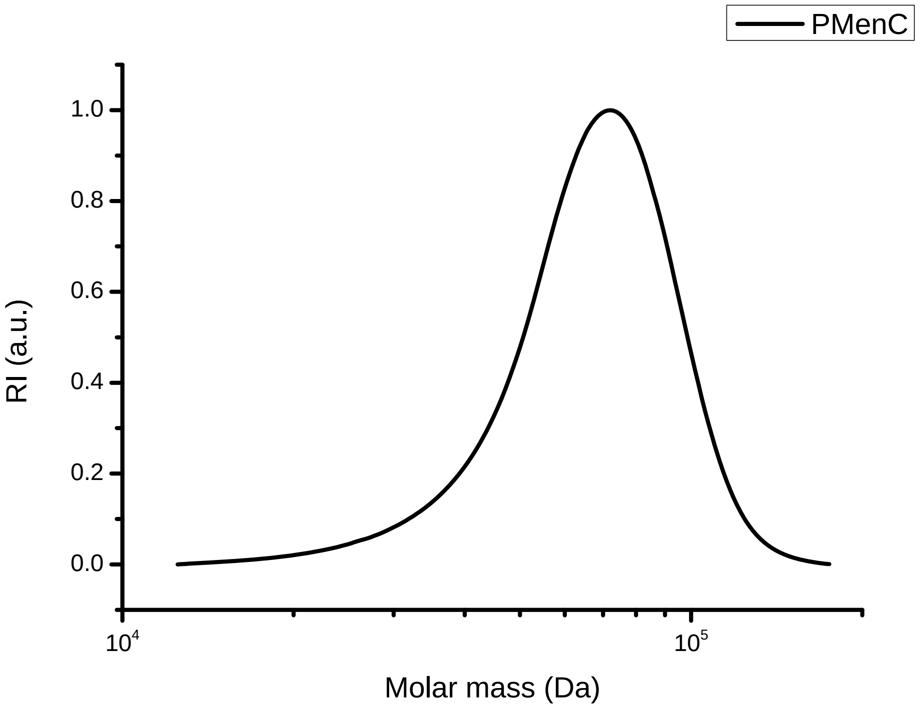

Supplementary Figure 28: GPC molar mass distribution (eluent was chloroform, calibration was performed with PS standards) of PMenC. The GPC analysis givs a relative M_n of 61.3 kDa and Đ of 1.14.

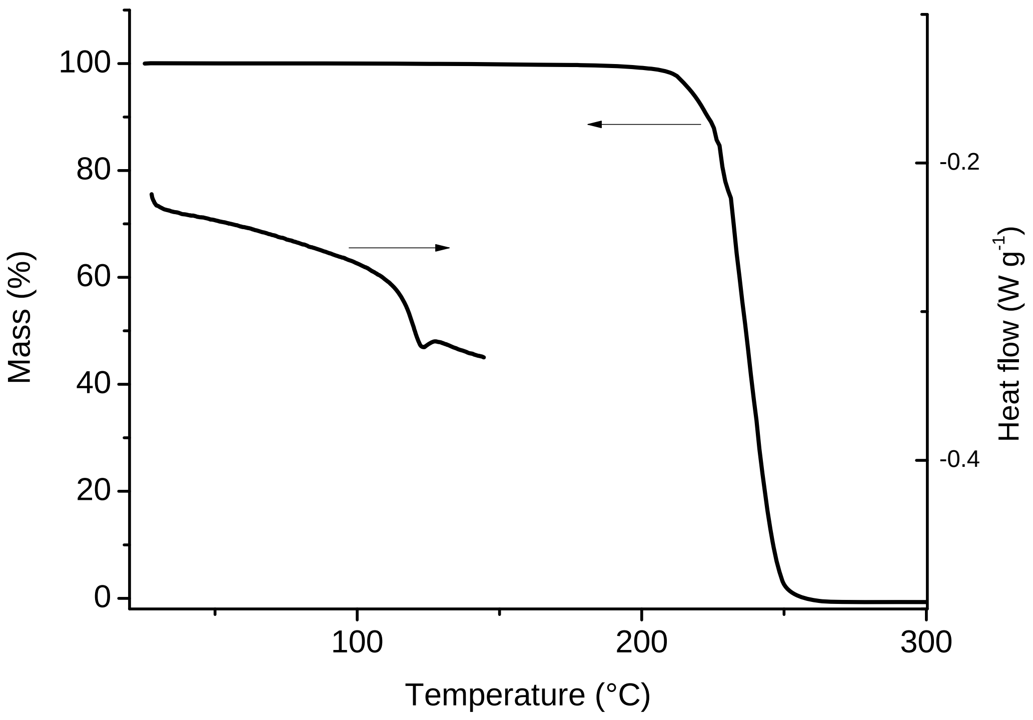

Supplementary Figure 29: TGA and DSC (2nd heating cycle) thermograms of PMenC measured at 10 K min^{-1} in N_2 atmosphere. The glass transition temperature is –as for PLimC - found at 130 °C and also the 5% decomposition temperature remains at 240 °C. Hence the hydrogenation has no influence on the thermal properties of this aliphatic polycarbonate.

Supplementary Table 1: Mechanical properties of selected commodities, engineering plastics and rubbers compared to PLimC and PLimC rubber from selected sources.[1–3]

Polymer	E Modulus [GPa]	σ_s [MPa]	ε [%]
PLimC	0.95	55	15
PLimC $B3MP_{2.0}$	0.001	6.8	228
PA-6	1.9	50	300
PC	2.5	65	125
PE	0.2 - 1	10 -30.0	600 - 800
PET	3	54	275
PMMA	3.2	65	10
PP	1.4	32	400
PTFE	0.5	13	200
PVAc	0.6	30	10 - 20.0
PVC	2.6	48	30
Silicon rubber	0.001	4.8 - 7.0	100 - 400
natural rubber	0.0013	17-25	750-850
1,4-Polybutadiene	0.0013		
butyl rubber	0.001	18-21	750-950
Neoprene	0.0016	25	800-1000

Supplementary Table 2: Functionalization of PLimC with B3MP for various reaction times, resulting in different DFs and therefore different amounts of residual double bonds. The mol% of unsaturation of the uncured sample (100% - DF) is given as subscript in the sample name (i.e. PLimC-B3MP$_{2.0}$ has 2 mol% unsaturated repeating units). The mechanical data was recorded after curing the samples for 5 h at 100 °C in air.

#	Sample	t [min]	DF_{B3MP}[a)] [mol%]	M_n[b)] [kDa]	Đ[b)]	σ_s[c)] [MPa]	Young's Modulus[c)] [MPa]	ε[c)] [%]
**1**	PLimC	0	0	54.3	1.13	950	55	15
**2**	PLimC-B3MP$_{2.0}$	60	98.0	72.3	1.18	6.8 ± 0.8	1.05 ± 0.12	228 ± 6
**3**	PLimC-B3MP$_{1.0}$	80	99.0	73.3	1.18	7.2 ± 2.8	0.90 ± 0.15	265 ± 8
**4**	PLimC-B3MP$_{0.5}$	180	99.5	75.2	1.18	9.0 ± 0.9	0.52 ± 0.05	342 ± 9
**5**	PLimC-B3MP$_{0.0}$	300	>99.9	76.1	1.17	5.2 ± 0.2	0.32 ± 0.02	426 ± 4

a) determined/estimated from analysis of ^{1}H-NMR spectra in the region of 4.7 ppm (protons of double bond), b) determined by GPC analysis, c) determined by tensile tests.

Supplementary Table 3: Functionalization of PLimC with 2-(diethylamino)ethanethiol in chloroform at 60 °C for different reaction times and either 6 (entry 1) or 7 (entries 2-4) equivalents of thiol with respect to PLimC double bonds.

#	sample	t [h]	eq. thiol	DF_{amine} [%]	pencil hardness
**1**	PLimC-N23[a)]	3	6	23	7B
**2**	PLimC-N37	3	7	37	7B
**3**	PLimC-N46	4	7	46	8B
**4**	PLimC-N67	6	7	67	8B

[a)]molecular weight (M_n) of starting material PLimC was 57.3 kDa

Supplementary Table 4: Quaternization of PLimC-N with benzyl bromide for different degrees of amination. Reaction was conducted in neat benzyl bromide (3 eq.) for 24 h without stirring, since the mixture was too viscous. After dilution with THF the polymer solution was repeatedly precipitated in hexane.

#	sample	DF_{amine} [%]	$DF_{quaternized}$ [%]	pencil hardness
**1**	PLimC-NQ20	3	20	2B
**2**	PLimC-NQ37	0	37	2B
**3**	PLimC-NQ46	0	46	3B
**4**	PLimC-NQ61	6	61	3B

Supplementary Table 5: Shaking flask test of PLimC and PLimC-NQ20 (duplicate test) in buffer. In the table the colony forming count (cfu) after the specified time of inoculation is listed, recalculated from the 10-fold diluted sample. Besides the samples also positive reference (water soluble polyguanidine) was tested, to compare the antibacterial activity of PLimC-NQ.

**time**	**[cfu]**	**[cfu]**	**R [%]**	**[cfu]**	**R [%]**	**[cfu]**	**R [%]**
h	PLimC	PLimC-NQ20		PLimC-NQ20		positive Ref (PHMG)	
0	170	160	0	180	0	230	0
6	170	160	0	180	0	0	100
12	1870	370	80	260	86	0	100
24[a]	19000	660	97	2000	90	0	100
48[a]	100000	12300	88	10200	90	0	100

[a)]estimates are given, as the number of colonies even for the 10-fold dilution was above 300.

Supplementary Table 6: Repetition of shaking flask test of PLimC and PLimC-NQ20 (tested in total three times).

**time**	**[cfu]**	**[cfu]**	**R [%]**	**[cfu]**	**R [%]**
h	PLimC	PLimC-NQ20		positive ref. (PHMG)	
6	250	280	0	0	0
12	150	260	0	0	100
24	2000	15	99.3	0	100

Supplementary Table 7: Agar plates from spread bacteria suspensions of shaking flask test of PLimC, PLimC-NQ20 and PLimC-NQ37 for 6, 12, 24 and 48 h incubation time. A sample of PLimC-NQ37 was also tested in this run and interestingly, the antibacterial activity was lower compared to the less functionalized samples PLimC-NQ20. We would ascribe this rather unexpected behavior to the higher surface roughness of the cast films of PLimC-NQ37, leading to higher hydrophobicity although more polar groups are grafted to the polymer. Thus interaction with bacteria, i.e. with the cell membrane, is limited, which leads to low antibacterial activity.

**Incubation**	**6 h**		
**Concentration**	**10^0**	**10^{-1}**	
	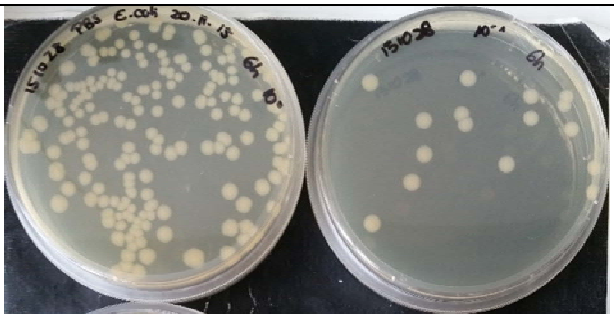		**PLimC-NQ20**
	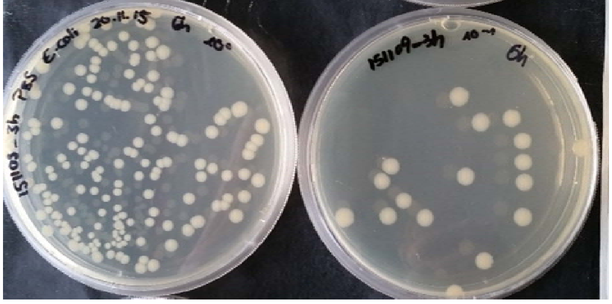		**PLimC-NQ37**
	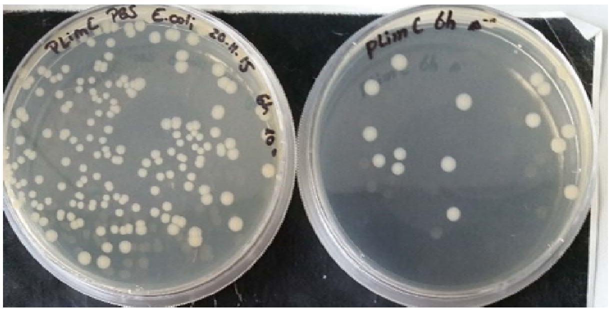		**PLimC**
	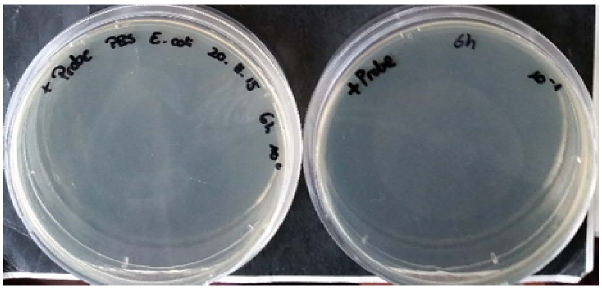		**Positive reference (PHMG)**

Supplementary Table 7 continued

Incubation	12 h		
**Concentration**	**10^0**	**10^{-1}**	
	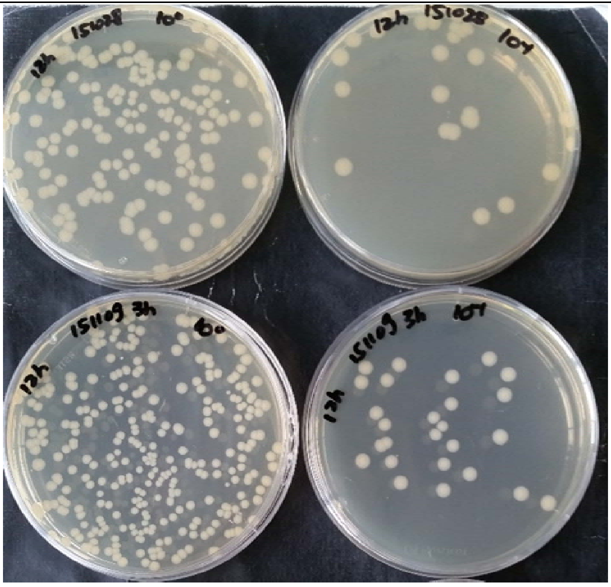		**PLimC-NQ20**
			**PLimC-NQ37**
	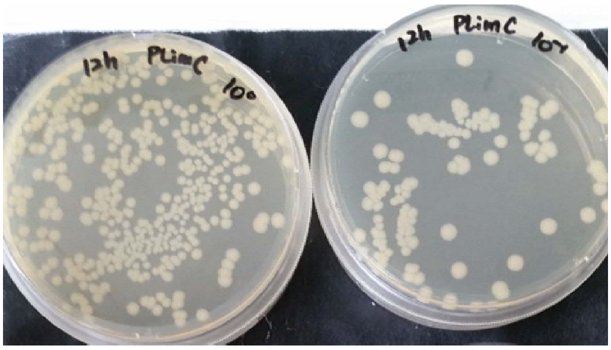		**PLimC**

Supplementary Table 7 continued

**Incubation**	**24 h**		
**Concentration**	**10^0**	**10^{-1}**	
	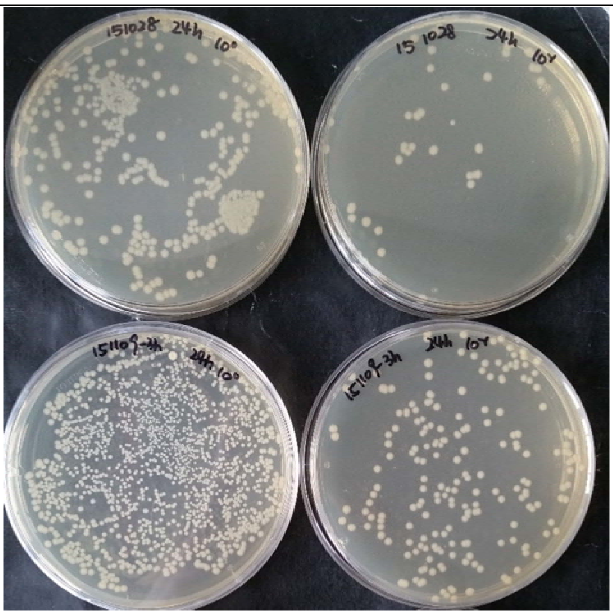		**PLimC-NQ20**
			**PLimC-NQ37**
	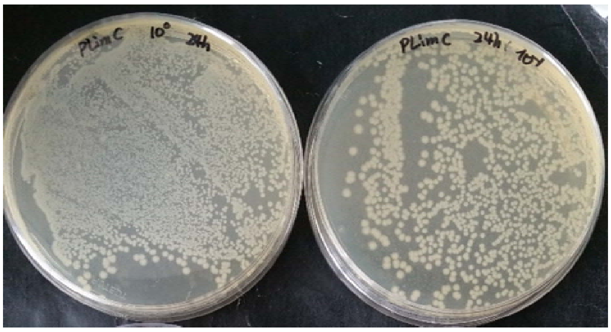		**PLimC**

**Incubation**	**48 h**		
**Concentration**	**10^0**	**10^{-1}**	
	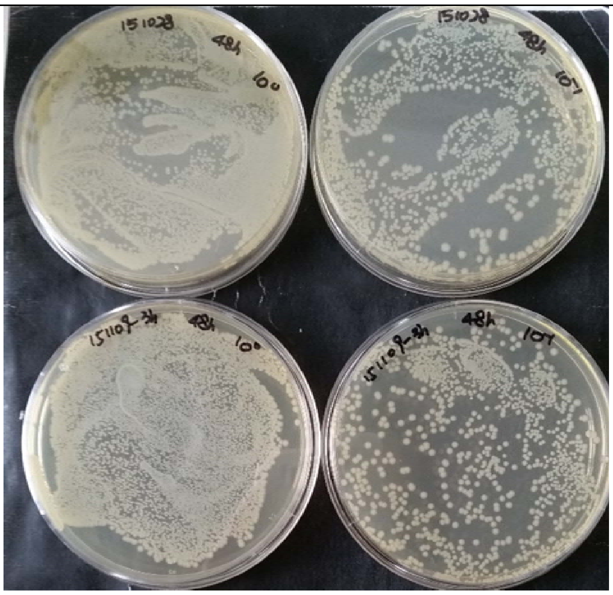		**PLimC-NQ20**
			**PLimC-NQ37**

Supplementary Table 8: Functionalization of PLimC with mercaptoethanol (PLimC-ME) for various reaction times, resulting in different degrees of functionalization and therefore lowered T_g and contact angles to water with respect to pure PLimC. The increasing T_g and CA_W for samples reacted for longer than 6 h, can be explained by cross-linking reactions between the double bonds, as the chain segment mobility is lowered and the cast films show a higher roughness, due to imperfections on the surface.

#	sample	t [h]	DF_{ME} [mol%]	M_n [kDa]	Đ	T_g [°C]	CA_W [°]
**1**	PLimC	0	0	42.1	1.13	130	94
**2**	PLimC-ME7	1	7	42.9	1.08	113	86
**3**	PLimC-ME18	2	18	49.2	1.08	110	80
**4**	PLimC-ME46	3	46	56.5	1.06	100	79
**5**	PLimC-ME82	6	82	63.4	1.11	91	70
**6**	PLimC-ME81	16	81	70.5	1.26	95	97[a)]

[a)]The prolonged reaction times lead to cross-linking, which also influenced surface roughness of cast films and thus the contact angle (higher for increased roughness).

Supplementary Table 9: Functionalization of PLimC by sulfuric acid-catalyzed electrophilic addition of PEG-3-OH for different reaction times.

#	sample	DF_{PEG}[%]	t [h]	T [°C]	CA_W[°]	M_n [kDa]	Đ	T_g [°C]
1	PLimC	-	-	-	93	54.3	1.10	130
2	PLimC-PEG0.7	0.7	23	20	94	53.2	1.12	
3	PLimC-PEG1.7	1.7	43	30	99	47.2	1.24	129
4	PLimC-PEG6.5	6.5	48	45	81	40.8	1.36	114
5	PLimC-PEG18.1	18.1	68	54	79	31.6	1.32	91

Supplementary Table 10: Weight loss studies of PLimC, PLimC-ME7/ME46/ME82 and BPA-PC in various media, i.e. water, esterase, pH and pH 3 buffer for 3, 7 and 21 days. All samples were measured in triplicate and the mean values are given here relative to the original mass in % with standard deviation σ. The change of hydrophilicity and T_g do not promote mass loss of the polymer samples PLimC, PLimC-ME7/46/82 or BPA-PC respectively, for the conditions tested.

		**PLimC**		**PlimC-ME7**		**PLimC-ME46**		**PLimC-ME82**		**BPA-PC**	
**medium**	**time [d]**	**mass [%]**	**σ**	**mass [%]**	**σ**	**mass [%]**	**σ**	**mass [%]**	**σ**	**mass [%]**	**σ**
**water**	3	98.0	0.3	99.0	0.3	98.8	0.3	98.5	0.5	98.5	0.5
	7	97.8	0.2	98.6	0.7	99.0	0.2	98.8	0.6	99.0	0.2
	21	98.6	0.7	98.8	0.1	98.5	0.6	98.5	0.4	99.3	0.1
**Esterase**	3	98.2	0.2	98.8	0.6	97.8	0.5	98.0	0.9	98.5	0.4
	7	99.1	0.0	98.9	0.2	98.3	0.3	99.0	0.5	98.8	0.3
	21	98.0	0.8	99.4	0.3	98.7	0.5	98.8	0.1	97.9	0.8
**pH 9**	3	98.4	0.5	99.5		98.5	0.5	98.5	0.5	98.8	0.3
	7	99.1	0.4	99.2	0.1	98.3	0.6	97.8	1.0	99.0	0.2
	21	98.9	0.4	98.9	0.6	99.1	0.1	98.6	0.4	98.5	0.6
**pH 4**	3	99.4	0.3	99.0	0.5	98.5	0.5	98.5	0.3	99.3	0.4
	7	98.6	0.7	98.1	0.7	98.8	0.2	98.8	0.8	98.5	0.8
	21	98.8	0.1	98.3	0.1	97.9	0.6	98.0	0.6	98.9	0.3

Supplementary Discussion

GPC analysis of PLimC-MAc

GPC data of the acid-functionalized PLimC sample (PLimC-MAc) is not given as it is not soluble in $CHCl_3$ and THF (only swells the polymer). The polymer is soluble in DMSO and DMF, though the sample elutes within the exclusion volume of the GPC column set due to the strong interaction with the eluent and weak interaction with the hydrophobic column packing material.

Curing procedure and tensile tests for PLimC rubber

The mechanical properties listed in Supplementary Table 2 were measured on samples that had been cured at 100 °C for 5 h in air. The curing procedure was applied in order to induce cross-linking of the residual double bonds in the polymer. After curing, the samples were assessed towards their solubility in chloroform and except for PLimC-B3MP$_{0.0}$ (no cross-linking is possible even when exposed to 100 °C for 24 h) none of them dissolved but only swelling was observed.

To elucidate the mechanism of heat-induced cross-linking, a solution of PLimC-B3MP$_{2.0}$ in chloroform was mixed with 1 wt% butylated hydroxytoluene (BHT, radical inhibitor) and cast into a film. After exposing the dry film to the same curing procedure (100 °C for 5 h) – in contrast to the BHT-free samples – no cross-linking was observable, indicating a free radical cross-linking mechanism between adjacent double bonds of the partially unsaturated samples. The heat-induced cross-linking was proven indirectly by assessing the solubility and the mechanical properties of the cured samples. A direct analysis of residual double bonds by IR spectroscopy is not applicable because the low degree of unsaturation (< 3%) lies beyond the detection limit of the spectrometer. The effect of curing (time) is shown in Supplementary Fig. 5 for PLimC-B3MP$_{2.0}$. The curing temperature of 100 °C was chosen due to the trade-off between decomposition of the backbone of the rather labile polycarbonate (as discussed in our previous article on PLimC)[3] and the rate of thermally induced cross-linking (the higher the faster). On the one hand, a curing time of 5 h was found to be sufficient, since longer curing would not change the tensile properties any further. On the other hand, a curing time of 5 h was necessary, since samples heated for only 3 h were still soluble in chloroform. As an initial statement regarding the shelf life of PLimC-B3MP$_{2.0}$, we can state that a 100 µm film exposed

to ambient conditions (21 °C, air with 50% relative humidity, laboratory lighting) for 20 days is still soluble in chloroform.

The mechanical properties of the cured samples of PLimC-B3MP are shown in Supplementary Fig. 6. As expected, the introduction of cross-links leads to an increase of Young's modulus and tensile strength while the strain at break is reduced. With increasing cross-linking density, i.e. higher amount of double bonds in the uncured sample, the strain at break is decreasing while the Young's modulus is increasing. The tensile strength σ_s is running through a maximum for low cross-linking density (PLimC-B3MP$_{0.5}$) but remains higher than in the saturated sample (PLimC-B3MP$_{0.0}$). Furthermore, a study of elasticity is shown in Supplementary Fig. 7, where a cured PLimC-B3MP$_{2.0}$ sample was strained by 20% in 40 cycles (5 s strain, 10 s relaxation, strain rate 10 mm min^{-1}). The strain is not completely reversible (maximum stress drops from 0.08 MPa for each consecutive cycle down to 0.06 MPa for the last cycle), as the T_g (5 °C) of the polymer is very close to the testing temperature of 21 °C, i.e. the slow dynamics can be assigned to the slow segmental motion of the polymer backbone. To improve reversibility in the strain experiment, the modification of PLimC with thiols that lower the T_g well below 0 °C should be used.

Degradation tests in composting environment

The PLLA samples readily disintegrated within the first two weeks (holes appeared after 8 days), whereas PLimC samples did not show any traces of degradation even after 60 °C days under the conditions mentioned, i.e. no holes, no surface changes overserved by SEM imaging, no change of molecular weight (distribution) measured by GPC. From those observations we concluded, that PLimC possesses a rather good bio-stability against the industrial composting environment.

Degradation tests in enzymatic environment

The enzyme (13 000 units mL^{-1}, substrate: glyceryl tributyrate) was chosen because of its high activity in the cleavage of ester linkages of condensed matter like water-insoluble polyesters that have been synthesized and readily degraded with this enzyme in our group.[4] The change of hydrophilicity and T_g do not promote mass loss of the polymer samples PLimC, PLimC-ME7/46/82 or BPA-PC respectively, for the conditions tested (see Supplementary Table 8). Furthermore, for none of the samples a significant change in molar mass was observed (see GPC data in Supplementary Fig. 22), from which was deduced that the investigated polymers are stable under those conditions within 21 days. For further studies either the testing time

should be increased or harsher conditions have to be applied. BPA-PC was employed as reference material and so far it can be stated, that PLimC and its modifications exhibit similar stability under those conditions.

The saturation of PLimC

The conversion of the monomer MenO and pre-monomers menth-1-ene and MenBrOH, respectively, were monitored by GC analysis. The chromatograms combined with the peak information are shown in Supplementary Figs. 23-25, respectively. The GC analysis of the precipitation bath of PMenC (Supplementary Fig. 26) is added to prove the preferential incorporation of *trans*-MenO into the polymer chain. The accumulation of *cis*-MenO after polymerization is obvious, rising from 8% before to 56% after copolymerization with CO_2. The preferential incorporation is also represented in the NMR spectra of PMenC. The ^{1}H-NMR spectrum (Supplementary Fig. 27) shows a single peak at 5.00 ppm without any downfield shoulder, which would be an indication of incorporation of the *cis*-isomer into the backbone. This argument is further supported by the ^{13}C-NMR spectrum of PMenC, which shows only one carbonyl resonance at 152.2 ppm i.e. no stereo-irregularities are present. GPC analysis revealed a relative M_n of 61.3 kDa and Đ of 1.14 (Supplementary Fig. 28).

Supplementary Methods

Materials

(R)-(+)-Limonene (97%, Sigma-Aldrich), hydrogen (5.0, Linde Gase), *N*-bromosuccinimide (97%, Sigma-Aldrich), sodium hydride (60% dispersion in mineral oil), iodomethane (99%, stabilized with silver), 5% platinum on charcoal (99%), mercaptoethanol (99%, Sigma-Aldrich), mercaptoacetic acid (99%, Sigma-Aldrich), 2-(diethylamino)ethanethiol hydrochloride (95%, Sigma-Aldrich), butyl 3-mercaptopropionate (98%, Sigma-Aldrich), benzyl bromide (99%, Alfa Aesar), poly(ethylene glycol) monomethyl ether (97%, Sigma-Aldrich) were used as received. Azobisisobutyronitrile (AIBN) was recrystallized from methanol. Toluene was dried over *sec*-butyl lithium and distilled. Tetrahydrofuran was dried over CaH_2 and distilled, further dried over potassium and distilled before use. Chloroform was dried over CaH_2 and distilled. Carbon dioxide (5.0, Linde Gase) was dried by passing through a column packed with a molecular sieve of 3 Å. The zinc catalyst with β-diiminate (bdi) and acetate ligand [(bdi)Zn(μ-OAc)][5] and PLimC[3] were synthesized according to literature procedures.

Synthetic Procedures

All synthetic manipulations were carried out under exclusion of air in dry conditions, if not otherwise stated. The acid-catalyzed electrophilic addition of PEG-3-OH to PLimC and thiol-ene chemistry are polymer analogous reactions. However, the hydrogenation of the *exo* double bond of limonene was performed on the pre-monomer, which was subsequently epoxidized and copolymerized with CO_2, to give the polycarbonate PMenC.

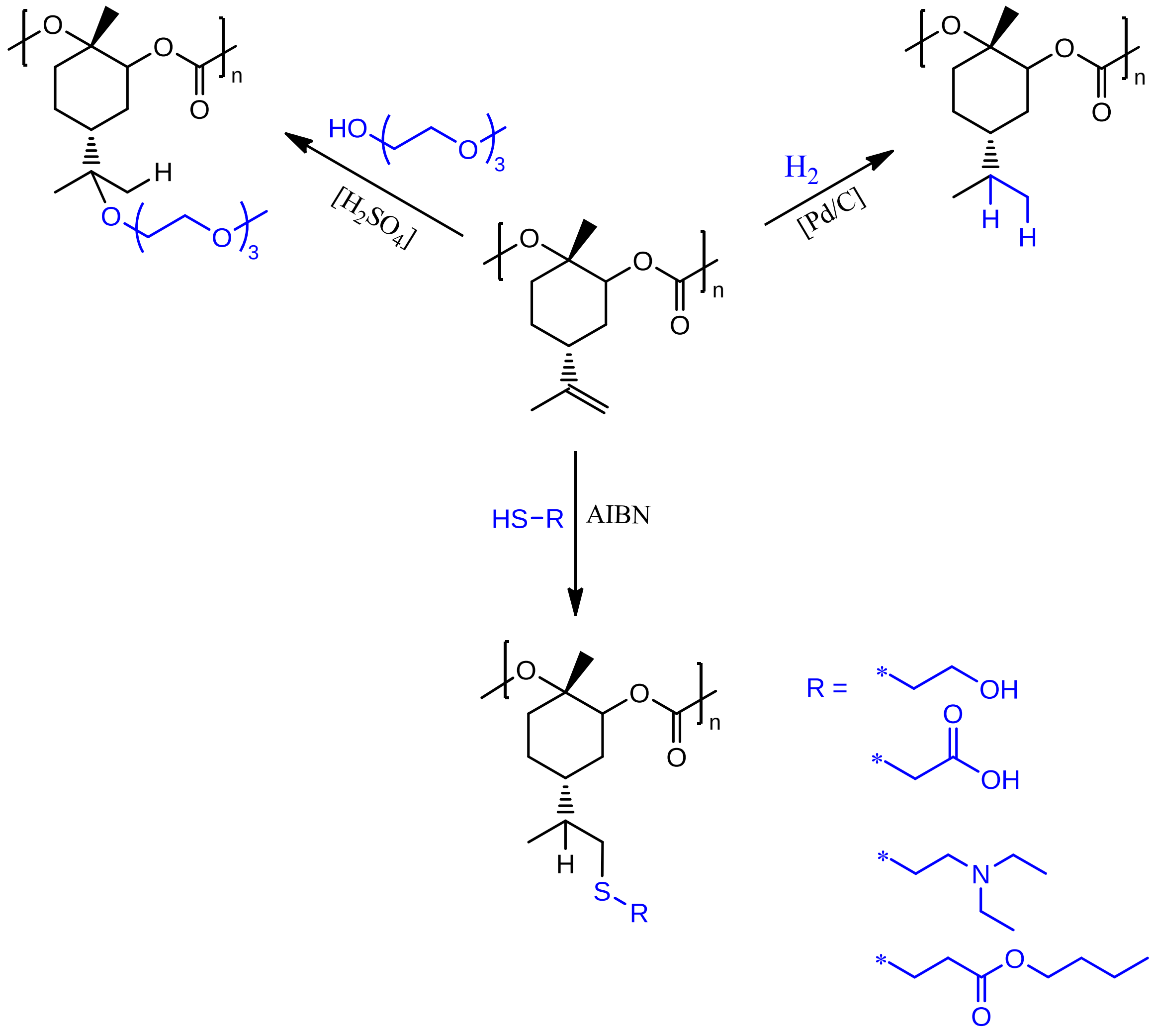

Thiol-ene chemistry on PLimC

PLimC was dissolved in degassed chloroform to produce a 2 wt% solution. After addition of 5 to 40 eq. of the desired thiol, 0.3 eq of AIBN were added. The solution was kept at 60 °C for the desired time, before the solution was concentrated and precipitated in an adequate non-solvent, corresponding to the functionalization, washed and reprecipitated when necessary. The resulting colorless samples were dried at 60 °C in vacuo (except for butyl 3-

mercaptopropionate functionalized PLimC that was dried at 20 °C and 0.02 mbar and stored in argon atmosphere).

PLimC-MAc: Functionalization with **mercaptoacetic acid**:

The solvent was removed *in vacuo* and the polymer redissolved in acetone before it was precipitated in water several times. The product was functionalized with 100% mercaptoethanol.

^{1}H NMR (300 MHz, DMSO-d^{6}): δ 5.01 (1H, s, C*H*O), 3.36 (2H, s, SC*H*$_2$CH), 3.16 (2H, s, SC*H*$_2$COOH), 2.62 (1H, m, SCH$_2$C*H*), 2.36 (1H, m, CH^{a}*H*^{b}COC=O), 2.22 (1H, m, SCH$_2$CHC*H*), 1.90 – 0.95 (8H, m, C*H*$_3$CC*H*^{a}H^{b}C*H*$_2$CHCC*H*$_2$), 0.85 (3H, s, C*H*$_3$CHCH) ppm

^{13}C NMR (300 MHz, DMSO-d^{6}): δ 171.6 (*C*OOH), 152.0 (*C*O$_3$), 81.5 (O*C*CH$_3$), 74.6 (*C*HO), 65.0 (*C*H$_2$COOH), 33.7, 33.6, 21.3, 15.5, 15.4, 15.2 ppm

PLimC-ME: Functionalization with **mercaptoethanol**:

The polymer was precipitated in a 1:1 mixture of methanol:water. The product was functionalized with up to 73% mercaptoethanol.

^{1}H NMR (300 MHz, CDCl$_3$): δ 5.04 (1H, s, C*H*O$_{\text{unfunctionalized}}$), 4.99 (1H, s, C*H*O$_{\text{functionalized}}$), 4.71 (2H, d, C=C*H*$_2$, ^{2}J=7.4 Hz), 3.70 (2H, m, CH$_2$C*H*$_2$OH), 2.68 (2H, m, C*H*$_2$CH$_2$OH), 2.55 (1H, m, SCH$_2$C*H*), 2.36 (1H, m, CH^{a}*H*^{b}COC=O), 2.21 (1H, m, CH$_3$CHC*H*), 1.90 – 1.00 (8H, m, C*H*$_3$CC*H*^{a}H^{b}C*H*$_2$CHCC*H*$_2$), 0.94 (3H, s, CHCHC*H*$_3$) ppm

^{13}C NMR (300 MHz, CDCl$_3$): δ 152.1, 148.7, 82.0, 75.4, 60.4, 37.3, 35.9, 34.0, 33.8, 21.7, 20.9, 16.0, 15.8 ppm

PLimC-B3MP: Functionalization with **butyl 3-mercaptopropionate**:

The polymer was precipitated in methanol and subsequently dried at 20 °C and 0.02 mbar to avoid cross-linking of residual double bonds. The polymer was stored in Ar atmosphere.

^{1}H NMR (300 MHz, CDCl$_3$): δ 4.96 (1H, s, C*H*O), 4.07 (2H, m, C*H*$_2$OH), 2.72 (2H, m, SC*H*$_2$CH$_2$), 2.56 (3H, m, C*H*C*H*$_2$S), 2.36 (2H, m, C*H*$_2$COC=O), 2.00 – 1.00 (14H, m, C*H*$_3$CCH$_2$C*H*$_2$C*H*CC*H*$_2$ / C*H*$_2$C(=O)OCH$_2$C*H*$_2$C*H*$_2$), 0.94 (6H, s, CHCHC*H*$_3$ / CH$_2$C*H*$_3$) ppm

^{13}C NMR (300 MHz, CDCl$_3$): δ 172.1, 152.0, 82.0, 75.5, 64.7, 37.7, 37.4, 37.3, 37.2, 35.0, 34.0, 30.7, 27.8, 27.7, 21.6, 19.2, 15.5, 13.8 ppm

PLimC-N: Functionalization with **2-(diethylamino)ethanethiol hydrochloride**:

The polymer was precipitated in a basic 2:1 mixture of methanol:water (sodium bicarbonate was added, to produce a pH of 8) and washed with slightly basic water.

^{1}H NMR (300 MHz, $CDCl_3$): δ 5.02 (1H, s, C*H*$O_{unfunctionalized}$), 4.96 (1H, s, C*H*$O_{functionalized}$), 4.69 (2H, d, C=CH_2, ^{2}J=7.2 Hz), 2.60 (2H, m, SC$H_2$$CH_2$), 2.54 (2H, m, CH_2CH_2N), 2.52 (2H, m, NC$H_2$$CH_3$), 2.37 (1H, m, $CH^{a}H^{b}$COC=O), 2.22 (1H, m, C*H*C=CH_2), 2.00 – 1.10 (8H, m, CH_3CC$H^{a}H^{b}$CH_2CHCCH_2), 1.01 (2H, t, NCH_2CH_3, ^{3}J=7.1 Hz), 0.94 (3H, s, CHCHCH_3, ^{3}J=6.2 Hz) ppm

^{13}C NMR (300 MHz, $CDCl_3$): δ 152.1, 148.7, 109.4, 82.1, 81.8, 75.4, 53.0, 47.1, 37.9, 37.5, 30.4, 30.3, 21.7, 20.8, 15.9, 15.6, 11.9 ppm

PLimC-NQ: Quaternization of PLimC-N with **benzyl bromide**:

3 eq. benzyl bromide were added to PLimC-N to give a highly viscous mixture. The reaction mixture was kept at 22 °C for 24 h before the polymer was precipitated repeatedly in hexane.

^{1}H NMR (300 MHz, DMSO-d^{6}): δ 7.52 (5H, m, C_6H_5), 5.05 (1H, s, C*H*$O_{unfunctionalized}$), 4.98 (1H, s, C*H*$O_{functionalized}$), 4.69 (2H, d, C=CH_2, ^{2}J=7.2 Hz), 4.62 (2H, m, C_6H_5CH_2), 3.27 (6H, m, N(C$H_2$$CH_2$)$_2CH_2$$CH_2$), 2.94 (2H, m, SC$H_2$CH), 2.64 (2H, m, SC$H_2$$CH_2$), 2.33 (1H, m, $CH^{a}H^{b}$COC=O), 2.13 (1H, m, C*H*C=CH_2), 2.00 – 1.00 (14H, m, CH_3CC$H^{a}H^{b}$CH_2C*H* (C*H*CH_3)CH_2), 0.84 (6H, m, N(CH_2CH_3)$_2$) ppm

Acid-catalyzed electrophilic addition of PEG-3 to PLimC

PLimC was dissolved in chloroform (1.3 wt% solution), before 12 eq. of PEG-3-OH and 0.33 eq. of concentrated H_2SO_4 were added. The reaction mixture was stirred for the desired time (24 – 68 h), concentrated *in vacuo* and the polymer precipitated in methanol. The colorless polymers were characterized by NMR/IR spectroscopy and contact angle measurements.

^{1}H NMR (300 MHz, $CDCl_3$): δ 5.04 (1H, s, CHO$_{unfunctionalized}$), 4.92 (1H, s, CHO$_{functionalized}$), 4.71 (2H, d, C=CH_2, ^{3}J=7.4 Hz), 3.63 – 3.45 (12H, m, PEG-3), 3.37 (3H, s, PEG-3-CH_3), 2.42 (1H, m, CH^aH^bCOC=O), 2.20 (1H, m, CHC=CH$_2$), 1.83 (2H, m, CHCH_2CH), 1.71 – 1.14 (9H, m, CH_3CCH^aH^{b}CH_2CHCCH_3), 1.06 (6H, s, CH$_2$OC(CH_3)$_2$) ppm

^{13}C NMR (300 MHz, $CDCl_3$): δ 152.0 (*C*O$_3$), 148.8 (C=*C*H$_2$), 109.4 (*C*=CH$_2$), 82.0 (O*C*CH$_3$), 76.2 (*C*OCH$_2$), 75.4 (*C*HO), 72.0 – 70.7 (PEG-3), 60.4 (PEG-3-*C*H$_3$), 59.2 (CO*C*H$_2$), 37.5 (*C*HC), 30.9 (CH$_2$*C*H$_2$C), 22.3 (CH*C*H$_2$CH), 21.6 (*C*H$_2$CH$_2$C), 21.0 (*C*H$_3$COC), 20.7 (*C*H$_3$C(CH)CH$_2$) ppm

The saturation of PLimC (PMenC)

Synthesis of menth-1-ene.[6] In a 130 mL stainless-steel autoclave 32 mL R-limonene and 0.05 mol% Pt (5wt% on C) were added. The autoclave was pressurized with 10 atmospheres of H_2 and the suspension was stirred for 11 h at 25 °C. After filtration through a G4 glass frit the regioselectively hydrogenated terpene was used for stereoselective epoxidation. The reaction was monitored by gas chromatography. The colorless liquid consisted of 3.6% *cis*-menthane, 3.0% *trans*-menthane, 86.9% menth-1-ene and 6.5% (R)-limonene (retention times: 14.33 (*cis*-menthane), 14.86 (*trans*-menthane), 16.17 (menth-1-ene), 16.36 min ((R)-limonene)).

Synthesis of trans-menth-1-ene oxide. The procedure for the stereoselective epoxidation of menth-1-ene is analogue to the stereoselective epoxidation of (R)-limonene described elsewere.[3,7] The epoxidation of menth-1-ene has also been subject in patent literature.[8] The final product was analysed by gas chromatography and consisted of 2.0 % *cis*-menthane, 2.0 % *trans*-menthane, 8.5% *cis*-MenO and 85 % *trans*-MenO and 6.5 % by-products (retention times: 14.85 (cis-menthane), 20.87 (*cis*-MenO), 21.03 min (*trans*-MenO).

Masking of hydroxyl impurities in menth-1-ene oxide. Hydroxyl-containing impurities were masked according to a procedure previously described.[3] The product was purified by vacuum distillation. The purified product used for polymerization consisted of 0.9 % *cis*-menthane, 1.0 % *trans*-menthane, 9.0% *cis*-MenO and 88.9 % *trans*-MenO.

Synthesis of poly(menthene carbonate). The polymerization was carried out in accordance to the copolymerization of LO and CO_2 that was described elsewhere.[3] The product was characterized by ^{1}H and ^{13}C NMR spectroscopy. The precipitation bath was concentrated in vacuo and the residue analyzed by GC, whereas the chromatogram revealed an accumulation of *cis*-MenO, supporting the expectation that only the *trans*-isomer would be incorporated into the polymer.

^{1}H NMR (300 MHz, $CDCl_3$): δ 5.00 (1H, s, C*H*O), 2.35 (1H, m, $CH^aH^bCOC{=}O$), 1.95 – 1.00 (10H, m, $CH_3CCH^aH^bCH_2CH(CH_2)CH$), 0.85 (6H, m, $CH(CH_3)_2$) ppm

^{13}C NMR (300 MHz, $CDCl_3$): δ 152.0 (CO_3), 82.2 ($COCCH_3$), 75.7 (OCH), 36.3 ($CHCH(CH_3)_2$), 32.1 ($CHCH(CH_3)_2$), 30.7 (CH_2CH_2C), 23.8 ($CHCH_2CH$), 21.7 (CH_2CH_2C), 19.8 (CH_3CCH_3), 19.6 (CH_3CCH_3) ppm

Supplementary References

1. Fried, J. R. *Polymer Science and Technology, Third Edition*. (Prentice Hall, 2014).

2. Brandrup, J., Immergut, E. H. & Grulke, E. A. *Polymer Handbook, Fourth Edition*. (Wiley, 1998).

3. Hauenstein, O., Reiter, M., Agarwal, S., Rieger, B. & Greiner, A. Bio-based polycarbonate from limonene oxide and CO_2 with high molecular weight, excellent thermal resistance, hardness and transparency. *Green Chem.* **18,** 760–770 (2016).

4. Buchholz, V., Agarwal, S. & Greiner, A. Synthesis and Enzymatic Degradation of Soft Aliphatic Polyesters. *Macromol. Biosci.* **16,** 207–213 (2016).

5. Allen, S. D., Moore, D. R., Lobkovsky, E. B. & Coates, G. W. Structure and reactivity of mono- and dinuclear diiminate zinc alkyl complexes. *J. Organomet. Chem.* **683,** 137–148 (2003).

6. Newhall, W. F. Derivatives of (+)-Limonene. I. Esters of trans-p-Menthane-1,2-diol. *J. Org. Chem.* **23,** 1274–1276 (1958).

7. Gurudutt, K. N., Rao, S. & Srinivas, P. Stereospecific synthesis of trans-β-terpineol. *Flavour Fragr. J.* **7,** 343–345 (1992).

8. Leffingwell, J. C. & Shackelford, R. E. Verfahren zur Herstellung von 1-Menthenepoxyd. 1–12 (1967).
